# A TLR7/8 agonist increases efficacy of anti-fentanyl vaccines in rodent and porcine models

**DOI:** 10.1038/s41541-023-00697-9

**Published:** 2023-07-24

**Authors:** Bethany Crouse, Shannon M. Miller, Peter Muelken, Linda Hicks, Jennifer R. Vigliaturo, Cheryl L. Marker, Alonso G. P. Guedes, Paul R. Pentel, Jay T. Evans, Mark G. LeSage, Marco Pravetoni

**Affiliations:** 1 Department of Pharmacology, University of Minnesota Medical School, Minneapolis, MN USA; 2 Department of Veterinary Population Medicine, University of Minnesota, St. Paul, MN USA; 3 Department of Biomedical and Pharmaceutical Sciences, Center for Translational Medicine, University of Montana, Missoula, MT USA; 4 Inimmune Corporation, Missoula, MT USA; 5 Department of Medicine, Hennepin Healthcare Research Institute, Minneapolis, MN USA; 6 Department of Veterinary Clinical Sciences, University of Minnesota, St. Paul, MN USA; 7 Department of Psychology, University of Minnesota, Minneapolis, MN USA; 8 Center for Immunology, University of Minnesota Medical School, Minneapolis, MN USA; 9 Department of Psychiatry and Behavioral Sciences, University of Washington School of Medicine, Seattle, WA USA; 10 Center for Medication Development for Substance Use Disorders, Seattle, WA USA; 11 Present Address: HealthPartners Institute, Research and Evaluation Division, 8170 33rd Ave S, Bloomington, MN 55425 USA; 12 Present Address: Luvo Bioscience, 7500W. Henrietta Road, Rush, NY 14543 USA

**Keywords:** Adjuvants, Conjugate vaccines, Antibodies, Toll-like receptors, Addiction

## Abstract

Opioid use disorders (OUD) and overdose are public health threats worldwide. Widespread access to highly potent illicit synthetic opioids such as fentanyl is driving the recent rise in fatal overdoses. Vaccines containing fentanyl-based haptens conjugated to immunogenic carrier proteins offer a long-lasting, safe, and cost-effective strategy to protect individuals from overdose upon accidental or deliberate exposure to fentanyl and its analogs. Prophylactic or therapeutic active immunization with an anti-fentanyl vaccine induces the production of fentanyl-specific antibodies that bind the drug in the blood and prevent its distribution to the brain, which reduces its reinforcing effects and attenuates respiratory depression and bradycardia. To increase the efficacy of a lead anti-fentanyl vaccine, this study tested whether the incorporation of synthetic toll-like receptor (TLR) 4 and TLR7/8 agonists as vaccine adjuvants would increase vaccine efficacy against fentanyl challenge, overdose, and self-administration in either rats or Hanford miniature pigs. Formulation of the vaccine with a nucleolipid TLR7/8 agonist enhanced its immunogenicity and efficacy in preventing fentanyl-induced respiratory depression, analgesia, bradycardia, and self-administration in either rats or mini-pigs. These studies support the use of TLR7/8 adjuvants in vaccine formulations to improve their clinical efficacy against OUD and potentially other substance use disorders (SUD).

## Introduction

Opioid use disorders (OUD) and overdose are currently at epidemic levels in the United States, with over 2 million people meeting the diagnostic criteria for OUD^1^ and over 180 people dying every day from opioid overdose. In 2019, 72.9% of opioid overdoses were due to synthetic opioids such as fentanyl and its highly potent derivatives^2^. The rate of overdose due to synthetic opioids increased 15% from 2018 to 2019^2^, and it is estimated that the COVID-19 pandemic has further increased fatal opioid overdoses by over 30% in 2020^3^. In 2021, fatal drug overdose deaths exceeded 100,000 in the United States^4^, and that trend continued in 2022. While there are FDA-approved medication-assisted therapies (MAT) for OUD, such as the mu-opioid receptor (MOR) agonist methadone, MOR partial agonist buprenorphine, and MOR antagonist naltrexone, these therapies are relatively hard to access, have limited efficacy, cause unwanted side effects, or have an intrinsic abuse liability^5,6^. The MOR antagonist naloxone, approved for the reversal of overdose, has a relatively short duration of action and must be administered after an overdose occurs, limiting its use against highly potent or longer-lasting synthetic opioids or in situations where it is not immediately accessible^7^. Furthermore, as a MOR antagonist, naloxone does not reverse opioid-induced effects mediated by cholinergic and adrenergic receptors such as “wooden chest syndrome”, a stiffening of the chest wall muscles which may complicate the performance of emergency cardiopulmonary resuscitation (CPR)^8^. For these reasons, novel treatments for OUD and overdose are urgently needed to prevent deaths due to opioid misuse or accidental exposure.

One such intervention consists of active immunization with an anti-opioid conjugate vaccine. These vaccines consist of opioid-based small molecule haptens conjugated to larger immunogenic carriers, which are often formulated with an adjuvant such as aluminum hydroxide (alum) to increase the generation of opioid-specific polyclonal antibodies^9^. Anti-drug antibodies selectively bind to the target opioid in plasma and reduce the distribution of free (unbound) drug to the brain, attenuating opioid-induced effects such as, antinociception, respiratory depression, bradycardia, and potential lethality^10–23^. Given their selectivity for the target opioid, vaccines can be used therapeutically in combination with current treatments for OUD or overdose and could also be administered prophylactically to prevent overdose in high-risk populations^10–23^. These vaccines have been extensively tested in preclinical models for efficacy and safety^10–23^, and a candidate anti-oxycodone vaccine is currently being tested in a Phase Ia/Ib clinical trial (NCT04458545).

One major barrier to the translation of vaccines for OUD and other substance use disorders (SUD) is the production of drug-specific antibodies at a concentration high enough to result in a significant reduction in drug use or drug-induced toxic effects. Phase III trials of vaccines against cocaine and nicotine showed that a significant reduction in drug intake occurred in a subset of patients that had the highest anti-drug antibody concentrations; however, most of the immunized patients were not such “high responders” (i.e., did not achieve high enough antibody levels for efficacy)^24,25^. To improve translation from preclinical models to clinical use, strategies are needed to increase the number of “high responders” to drug-based conjugate vaccines. This requires understanding the basic immunology behind conjugate vaccine response to choose appropriate delivery platforms and adjuvants to optimize the immune response.

The production of high-affinity opioid-specific antibodies after immunization relies on CD4^+^ T cell-dependent B-cell activation and the formation of germinal centers in the lymph nodes and spleen^10,26,27^. Additionally, data suggest that a balanced Th_1_/Th_2_ response and the production of a combination of IgG_1_ and IgG_2a_ antibodies in mice leads to higher efficacy against opioid challenges after immunization^26,27^. To facilitate clinical translation, most anti-opioid vaccine formulations are adsorbed on aluminum hydroxide (alum)^9,20^; however, this adjuvant has been shown to induce a strong Th_2_ response and the production of almost exclusively IgG_1_^28^. Beyond alum salts, preclinical studies have tested anti-opioid vaccines in combination with a variety of adjuvants and immunomodulators including the TLR4 agonist MPLA^29^, TLR9 agonist CpG ODN 1826^30^, dsRNA^12^, Advax^31^, army liposome formulation (ALF)^21^, anti-IL-4 monoclonal antibodies (mAbs)^26,27^, anti-IL-13 mAbs^27^, anti-IL-2R mAbs^26^, anti-IL-7R mAbs^27^, Freund’s^29^, dmLT^32^, LTA1^32^, and MF59^33^ with varying degrees of success in increasing anti-opioid antibody titers and inducing the production of IgG_2a_. While potentially effective, many of these adjuvants are not feasible for clinical translation due to toxicity, side effects, cost, or availability due to intellectual property rights. In order to optimize the antibody response to a lead anti-fentanyl vaccine (F_1_-CRM, or F-CRM)^20,34,35^, our group tested the effect of adding two adjuvants targeting either TLR4 (INI-2002) or TLR7/8 (INI-4001) in mice (concurrent dual submission to NPJ Vaccines). Both TLR4 and TLR7/8 adjuvants have been shown to induce T cell responses and the production of IgG_2a_ antibodies when combined with protein-based or protein-conjugate vaccines^36–39^. Adjuvanting F_1_-CRM with an alum-adsorbed TLR7/8 agonist, but not a TLR4 agonist, increased the production of total IgG and specifically IgG_2a_ which led to an increase in efficacy after fentanyl challenge in mice (concurrent dual submission to NPJ Vaccines). Here, we extended these findings by testing this formulation in a rat model of fentanyl challenge, drug-induced toxicity, and fentanyl intravenous self-administration. Additionally, we tested whether the addition of INI-4001 would expand epitope recognition and increase cross-reactivity to off-target molecules. Finally, since rodent models do not fully recapitulate the human immune response after TLR7/8 activation^40–42^, a porcine model of fentanyl challenge and overdose was developed to provide preliminary evidence that this lead adjuvanted vaccine formulation is effective in large animal models that display TLR7/8-mediated activation representative of human immune responses. In these studies, an alum-adsorbed TLR7/8 agonist increased the immunogenicity and efficacy of a lead fentanyl vaccine in rat models of drug-challenge, overdose, and self-administration, as well as in a novel pig model of fentanyl overdose toxicity. This supports the use of INI-4001 as an adjuvant in clinical studies of the F_1_-CRM vaccine for the treatment of OUD and the prevention of opioid overdose.

## Results

### Activation of TLR7/8, but not TLR4, increases the efficacy of F_1_-CRM against fentanyl in rats

**Fig. 1:**
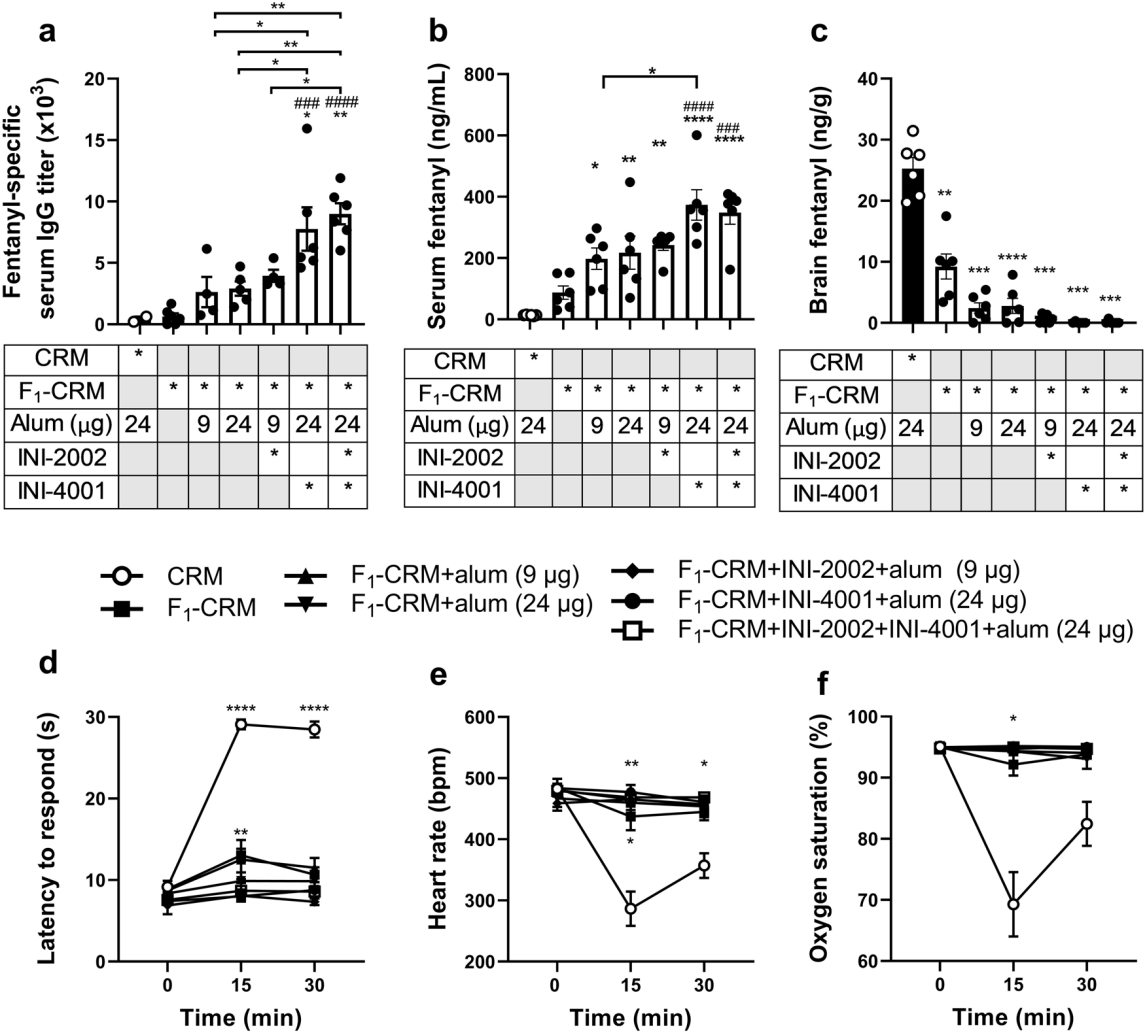
A TLR7/8 agonist, but not TLR4 agonist, increases efficacy of an anti-fentanyl vaccine in rats. Rats (*n* = 6/group) were immunized on day 0, 21, and 42. Blood was collected on day 49 for titer measurements. On day 56, rats were challenged with fentanyl (0.05 mg/kg), and parameters of vaccine efficacy were measured at 15 and 30 minutes. Following final behavioral measurements, blood and brain were collected to quantify fentanyl concentration. **a** Fentanyl-specific antibody titers on day 49. **b** Serum fentanyl concentration and **c** brain fentanyl concentration after drug challenge. **d** Latency to respond, **e** heart rate, and **f** oxygen saturation measured at two timepoints after drug challenge. Data are mean ± SEM. Statistical analysis was performed via one-way (**a**, **b**) or two-way (**d**–**f**) ANOVA with Tukey’s multiple comparisons post hoc test, or Brown–Forsythe and Welch ANOVA with Dunnett’s T3 multiple comparisons post hoc test (**c**) * directly over columns indicate significance compared to the control. ^#^ directly over columns indicate significance to F_1_-CRM alone. Statistical symbols: *P < 0.05, ** or ^##^*P* < 0.01, ^###^*P* < 0.001, **** or ^####^*P* < 0.0001.

Previous studies have shown that INI-4001 (TLR7/8 agonist), but not INI-2002 (TLR4 agonist), was effective at increasing fentanyl-specific IgG antibody titers and increasing vaccine efficacy against a fentanyl challenge in mice (data published in concurrent dual submission to NPJ Vaccines). To determine whether this effect was consistent between species and to provide proof of efficacy in more clinically relevant models of drug exposure, these findings were replicated by actively immunizing rats on days 0, 21, and 42 with F_1_-CRM alone, F_1_-CRM adsorbed on different doses of alum, F_1_-CRM and INI-2002 adsorbed on alum, F_1_-CRM and INI-4001 adsorbed on alum, or F_1_-CRM with a combination of all three adjuvants, mirroring the experimental design performed in mice (dual submission to NPJ vaccines). Analysis of fentanyl-specific serum IgG titers showed that groups immunized with the vaccine formulation containing INI-4001 reached significantly higher antibody titers (one-way ANOVA, *F*_(6,26)_ = 10.04) than groups immunized with CRM alone (Tukey’s, *p* = 0.0119), F_1_-CRM alone (Tukey’s, *p* = 0.0003), or F_1_-CRM with either 9 (Tukey’s, *p* = 0.0335) or 24 μg (Tukey’s, *p* = 0.0321) alum doses (Fig. 1a). Notably, the group immunized with all three adjuvants did not display increased titers compared to the F_1_-CRM+alum+INI-4001, indicating that TLR7/8 agonist activity was driving the increased antibody response. After immunization, rats were challenged with 0.05 mg/kg fentanyl. Latency to respond on a hot plate (Fig. 1d), heart rate (Fig. 1e), and oxygen saturation (Fig. 1f) were measured at baseline, 15 minutes, and 30 minutes after drug challenge. Analysis with two-way ANOVA with Geisser-Greenhouse correction shows that there was a significant main effect of time (*F*_(1.520, 53.18)_ = 61.43, *p* < 0.0001), vaccine group (*F*_(6, 35)_ = 60.00, *p* < 0.0001), and time x vaccine group interaction (*F*_(12, 70)_ = 21.90, *p* < 0.0001) on antinociceptive effects, a significant time (*F*_(1.845, 64.57)_ = 14.85, *p* < 0.0001), vaccine group (*F*_(6, 35)_ = 20.77, *p* < 0.0001) and time x vaccine group interaction (*F*_(12, 70)_ = 10.80, *p* < 0.0001) when assessing oxygen saturation, and a significant time (*F*_(1.947, 68.14)_ = 24.32, *p* < 0.0001), vaccine group (*F*_(6, 35)_ = 11.66, *p* < 0.0001), and time x vaccine group interaction (*F*_(12, 70)_ = 9.749, *p* < 0.0001) when assessing heart rate. Tukey’s multiple comparisons post hoc test indicates that almost all actively immunized groups had significantly decreased antinociception, bradycardia, and respiratory depression compared to the CRM immunized control in all parameters tested at 15 minutes (except for F_1_-CRM alone compared to CRM when measuring respiratory depression, Tukey’s, *p* = 0.0508), and antinociception and bradycardia were significantly decreased compared to control at 30 minutes, with the exception of the F_1_-CRM + 24 μg alum group compared to control when assessing bradycardia (Tukey’s, *p* = 0.0527). After the final measurement was taken, rats were euthanized and blood and brain were collected for analysis of opioid concentration by liquid chromatography/mass spectrometry (LC-MS). Groups adjuvanted with INI-4001 showed significantly increased serum fentanyl concentrations (one-way ANOVA, *F*_(6, 35)_ = 13.31) compared to the control (Tukey’s, *p* < 0.0001), F_1_-CRM alone (Tukey’s, *p* < 0.0001), or F_1_-CRM with 9 μg alum (Tukey’s, *p* = 0.0202), and trending increases in serum fentanyl compared to F_1_-CRM + 24 μg alum (Fig. 1b, *p* = 0.0534). Again, groups immunized with all three adjuvants did not show significantly different serum fentanyl concentrations as compared to the F_1_-CRM+alum+INI-4001 group, suggesting that INI-4001 is responsible for the increase in efficacy. Similar trends were found in brain fentanyl concentrations (Fig. 1c), where groups adjuvanted with INI-4001 displayed decreased brain fentanyl concentrations compared to all other groups (Brown-Forsythe and Welch ANOVA, F*_(6.000, 13.55)_ = 54.13), however, this was only significant compared to the CRM control (Dunnett’s T3, *p* = 0.0013). Overall, these results suggest that INI-4001, but not INI-2002, increases the efficacy of a lead fentanyl vaccine.

### Fentanyl vaccines adjuvanted with alum-adsorbed TLR7/8 showed increased protection against fatal levels of respiratory depression after fentanyl challenge

**Fig. 2:**
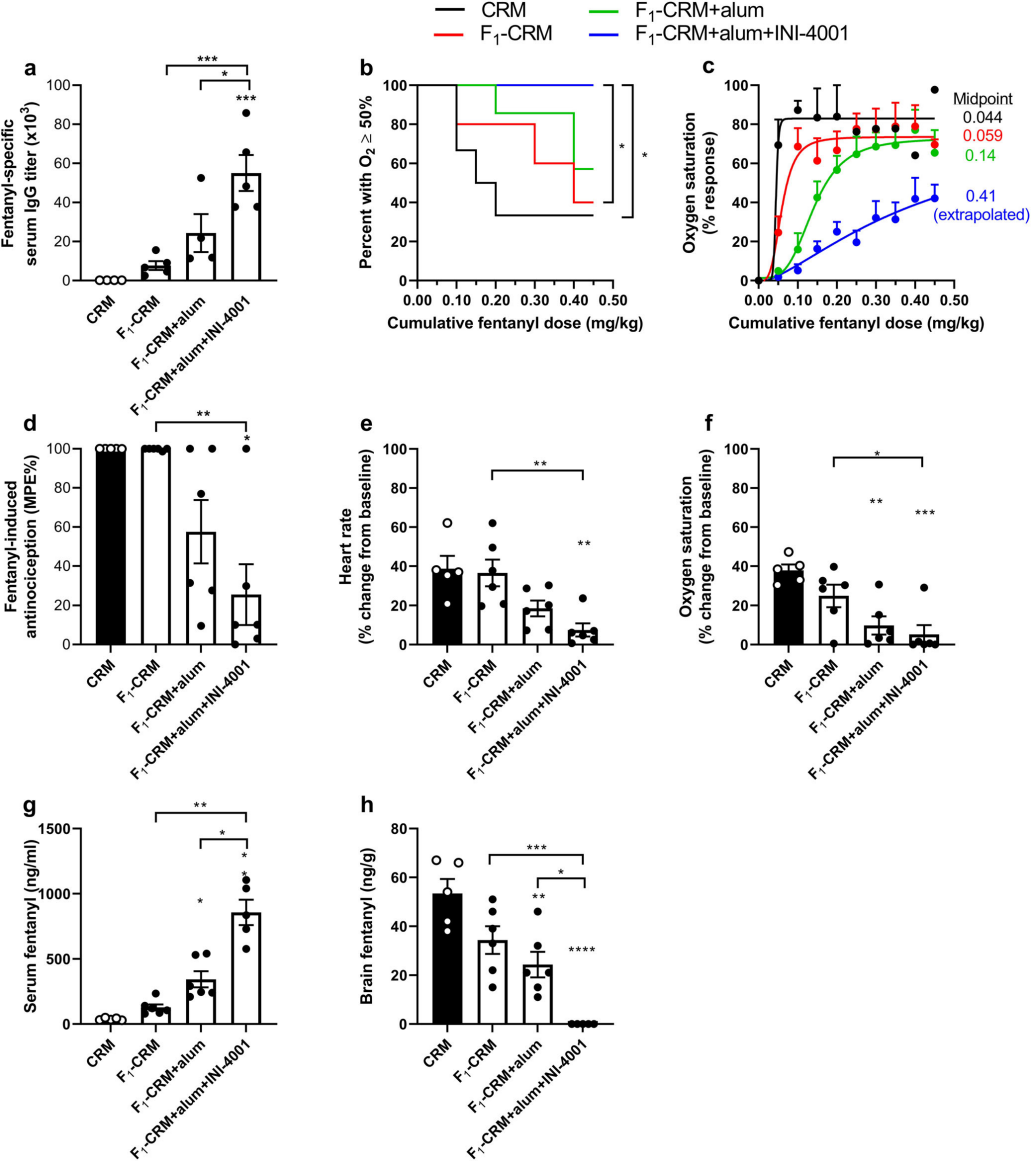
TLR7/8 adjuvanted vaccine protects against fatal levels of respiratory depression and high-dose fentanyl challenge. Rats (*n* = 6/group) were immunized on days 0, 21, and 42. On day 49, blood was collected to measure **a** fentanyl-specific IgG titers via ELISA. Starting on day 56, rats were challenged with a cumulative dose of fentanyl (0.45 mg/kg). Oxygen saturation was measured every 15 minutes. **b** Kaplan–Meier survival curve of rats who did not fall below 50% oxygen saturation as measured by pulse oximetry. **c** Dose–response curve of fentanyl-induced respiratory depression calculated as percent maximum possible response [(post-drug SaO_2_-baseline SaO_2_)/(SaO_2_ cutoff-baseline SaO_2_)]. The following week, rats were challenged with a bolus dose of fentanyl (0.1 mg/kg). 15 minutes post-challenge, **d** antinociception as a percent maximum possible effect, **e** heart rate as a percent change from baseline, and **f** oxygen saturation as a percent change from baseline was measured. After final measurement blood and brain were collected to determine **g** serum fentanyl concentration and **h** brain fentanyl concentration via LC-MS. Data are mean ± SEM. Statistical analysis was performed via one-way ANOVA with Tukey’s multiple comparison’s post hoc test (**a**, **e**, **f**, **h**), or Brown-Forsythe and Welch ANOVA with Dunnett’s T3 multiple comparisons post hoc test (**d**, **g**). Kaplan–Meier statistical analysis was performed via Mantel–Cox test between groups (**b**). * directly over columns indicate significance compared to the control. Statistical symbols: *P < 0.05, **P < 0.01, ***P < 0.001, ****P < 0.0001.

Since INI-4001, but not INI-2002, increased efficacy of a lead fentanyl vaccine, a follow-up experiment was performed where rats were immunized with CRM (negative control), F_1_-CRM (positive control), F_1_-CRM adsorbed on alum, or F_1_-CRM with INI-4001 adsorbed on alum. After three immunizations, rats immunized with the INI-4001 adjuvanted vaccine had significantly higher fentanyl-specific serum IgG antibody titers (one-way ANOVA, *F*_(3, 14)_ = 13.35) compared to all other groups, as expected (Tukey’s compared to CRM, *p* < 0.0003; compared to F_1_-CRM, *p* = 0.0007; compared to F_1_-CRM+alum, *p* = 0.0311; Fig. 2a). Rats were then challenged with repeated doses of 0.05 mg/kg fentanyl every 15 minutes, up to a 0.45 mg/kg cumulative dose. The rats’ oxygen saturation was monitored via pulse oximetry 15 minutes after each dose before immediately receiving the next dose. As prolonged periods of 50% oxygen saturation would likely be considered fatal in a human^43^, any rat whose oxygen saturation dropped below 50% was given naloxone and censored for the rest of the study. When assessing the number of rats that fell below 50% oxygen saturation using a Kaplan–Meier survival curve and Mantel-Cox test, rats immunized with F_1_-CRM+alum+INI-4001 displayed significantly increased survival from fatal levels of respiratory depression when compared to CRM (*p* = 0.0179) or F_1_-CRM alone (*p* = 0.0315, Fig. 2b) and trended towards increased survival compared to F_1_-CRM+alum (*p* = 0.0813). Notably, the group receiving the INI-4001 adjuvanted vaccine was the only group where none of the rats reached 50% oxygen saturation. When analyzing the change in oxygen saturation as a percent maximum possible response (baseline = 0% response and 50% oxygen saturation = 100% response), the group receiving the vaccine formulated with alum and INI-4001 displayed a midpoint value that was shifted 9.3×, 6.9×, and 2.9× compared to CRM control, F_1_-CRM alone, and F_1_-CRM+alum, respectively (Fig. 2c). The following week, the same rats were challenged with 0.1 mg/kg fentanyl, and antinociceptive response (Fig. 2d), heart rate (Fig. 2e), and oxygen saturation (Fig. 2f) were measured after the drug challenge. When compared to CRM or F_1_-CRM alone, rats receiving alum adsorbed INI-4001 adjuvanted vaccine had significantly decreased antinociceptive response (Brown-Forsythe and Welch ANOVA with Dunnett’s T3 multiple comparison’s *post hoc* test, F*_(3.000, 9.982)_ = 10.15; CRM *p* = 0.0224; F_1_-CRM *p* = 0.0227), bradycardia (one-way ANOVA with Tukey’s multiple comparison’s post hoc test, *F*_(3, 19)_ = 7.785; CRM *p* = 0.0037; F_1_-CRM *p* = 0.0045), and respiratory depression (one-way ANOVA with Tukey’s multiple comparison’s post hoc test, *F*_(3, 19)_ = 9.273; CRM *p* = 0.0008; F_1_-CRM *p* = 0.0357). Trending differences between F_1_-CRM+alum and F_1_-CRM+alum+INI-4001 were not yet significant, likely due to the individual variability in the pharmacological responses. Immediately after the last measurement, rats were euthanized, and blood and brain were collected to measure fentanyl concentrations. Rats immunized with F_1_-CRM+alum+INI-4001 displayed significantly increased serum fentanyl concentrations (Brown-Forsythe and Welch ANOVA with Dunnett’s T3 multiple comparison’s post hoc test, F*_(3.000, 7.910)_ = 36.94; CRM *p* = 0.0048; F_1_-CRM *p* = 0.0082; F_1_-CRM+alum *p* = 0.0153; Fig. 2g) and significantly decreased brain fentanyl concentrations when compared to all other groups (one-way ANOVA with Tukey’s multiple comparison’s post hoc test, *F*_(3, 18)_ = 17.99; CRM *p* < 0.0001; F_1_-CRM *p* = 0.0007; F_1_-CRM+alum *p* = 0.0148; Fig. 2h). Together, these data indicate that the F_1_-CRM + INI-4001+alum vaccine formulation produces a rightward shift in fentanyl’s dose-response curve and decreases fentanyl distribution to the brain, which increases protection from respiratory depression, bradycardia, and potential lethality from high doses of fentanyl.

### INI-4001 does not change IgG antibody specificity of the lead fentanyl vaccine

**Fig. 3:**
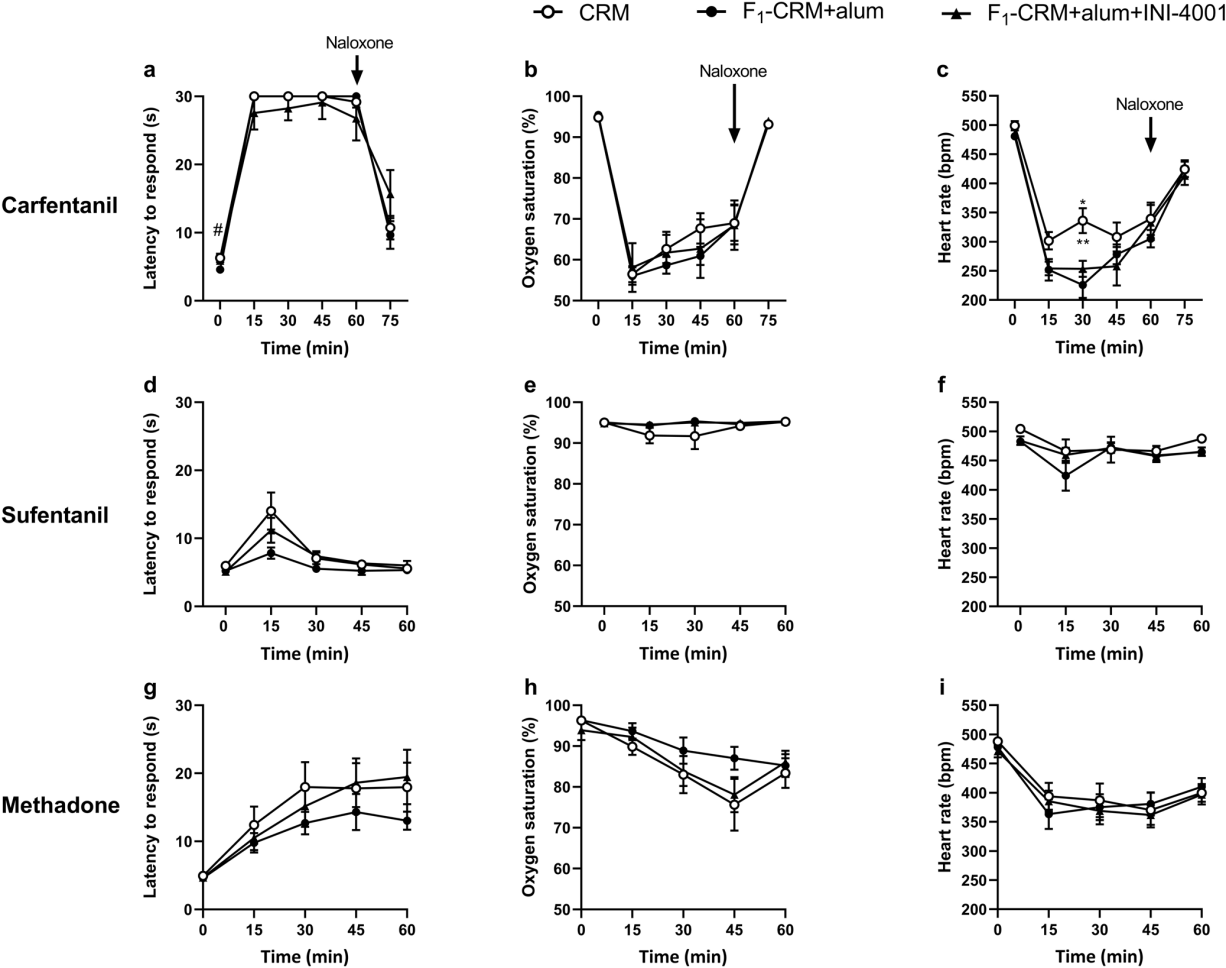
Addition of INI-4001 does not significantly alter in vivo efficacy of off-target molecules. Rats (*n* = 8/group) were immunized on days 0, 21, and 42. Starting on day 56, rats were challenged with one drug per week. **a** Latency to respond on a hot plate, **b** oxygen saturation, and **c** heart rate after challenge with carfentanil (0.01 mg/kg). 60 minutes post-challenge, rats were given naloxone (0.1 mg/kg) to reverse carfentanil-induced effects. **d** Latency to respond on a hot plate, **e** oxygen saturation, and **f** heart rate after challenge with sufentanil (0.008 mg/kg). **g** Latency to respond on a hot plate, **h** oxygen saturation, and **i** heart rate after challenge with methadone (4.5 mg/kg). Data are mean ± SEM. Statistical analysis was performed via two-way ANOVA with Geisser–Greenhouse correction and Tukey’s multiple comparisons post hoc test. * directly over columns indicate significance compared to the control. ^#^ directly over columns indicate significance to F_1_-CRM alone. Statistical symbols: * or ^#^*P* < 0.05, **P < 0.01.

One hypothesis regarding how adjuvants increase vaccine efficacy is that adjuvants can broaden the resultant vaccine-induced antibody diversity, expanding epitope recognition to protein antigens. To test whether this hypothesis also applied to INI-4001 adjuvanted vaccines against small molecule targets such as fentanyl, rats were immunized with F_1_-CRM+alum or F_1_-CRM+alum+INI-4001 to determine whether addition of INI-4001 would increase epitope diversity within the F_1_-specific antibody and B-cell repertoire that could either (1) expand cross-reactivity to additional fentanyl analogs or (2) increase cross-reactivity with off-target molecules such as naloxone, naltrexone, or methadone. Rats were first challenged with fentanyl in vivo after antibody titers were assessed via ELISA to ensure the adjuvant was effective at increasing anti-fentanyl vaccine efficacy (Supplementary Fig. 1). Afterward, rats were challenged with carfentanil followed by naloxone reversal (Fig. 3a–c), sufentanil (Fig. 3d–f), or methadone (Fig. 3g–i) on consecutive weeks. Antinociceptive response, oxygen saturation, and heart rate were monitored every 15 minutes for an hour after drug challenge. The addition of INI-4001 did not change the in vivo efficacy and selectivity of F_1_-CRM to any of the drugs tested, and notably did not interfere with naloxone reversal of carfentanil-induced respiratory depression. Serum samples were also tested for in vitro cross-reactivity to various fentanyl analogs and off-target medications (Table 1). While the specificity of the antibodies for fentanyl is high in both groups (IC_50_ < 15 nM), the addition of INI-4001 did not significantly change the affinity of the polyclonal antibodies for any of the drugs tested. These data suggest that addition of INI-4001 to an anti-fentanyl vaccine does not significantly change fentanyl epitope diversity and antibody binding which would lead to interference with other medications used to treat pain or OUD.

**Table 1:** Selectivity for fentanyl and lack of binding for off-target compounds (mean ± SEM).

	F_1_-CRM+alum		F_1_-CRM+alum+INI-4001	
Competitor	IC_50_ (μM)	Relative affinity	IC_50_ (μM)	Relative affinity
Fentanyl	0.014 ± 0.015	100%	0.0075 ± 0.0099	100%
Acetylfentanyl	0.200 ± 0.243	6.71%	0.100 ± 0.073	7.51%
Carfentanil	19.77 ± 22.12	0.068%	8.48 ± 3.49	0.089%
Alfentanil	91.29 ± 63.89	0.015%	207.57 ± 127.98	0.0036%
Remifentanil	121.45 ± 90.62	0.011%	384.59 ± 323.29	0.0020%
Sufentanil	319.16 ± 297.82	0.0042%	185.11 ± 286.9	0.0041%
Naltrexone	344.67 ± 201.67	0.0039%	821.00 ± 309.44	0.00092%
Naloxone	1877.37 ± 1788.03	0.00071%	3005.25 ± 1556.88	0.00025%
Buprenorphine	32.61 ± 22.07	0.04%	>50	<0.015%

Values are IC_50_ off-target compounds measured by competitive binding ELISA of serum antibodies after immunization against fentanyl.

### The fentanyl vaccine adjuvanted with INI-4001 and alum attenuates the reinforcing effects of fentanyl to a greater degree than vaccine with alum alone in intravenous self-administration models

**Fig. 4:**
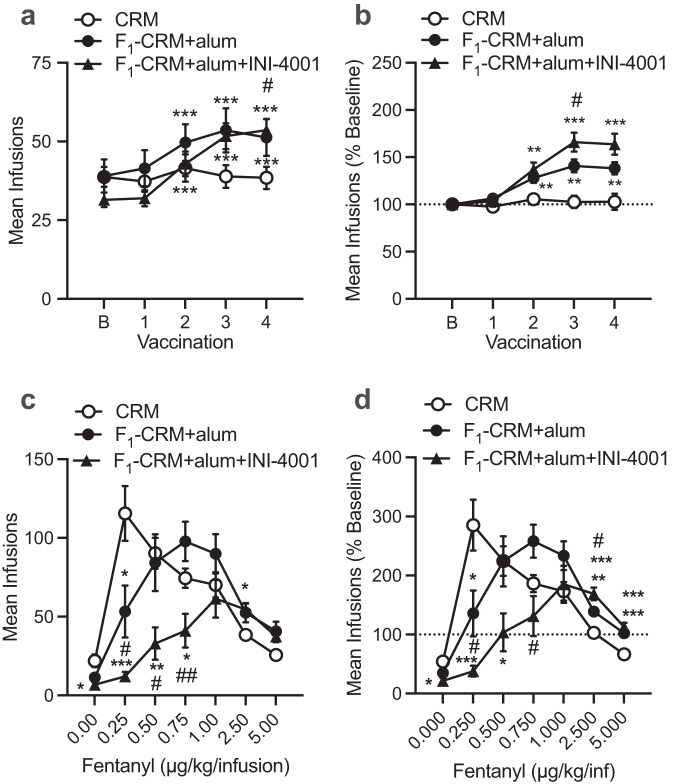
Effect TLR7/8 adjuvanted anti-fentanyl vaccine on maintenance of fentanyl self-administration. Mean (±SEM) fentanyl infusions in rats at baseline (B) and 2 weeks after each of the first four vaccinations with CRM (*n* = 11), F_1_-CRM+alum (*n* = 15), or F_1_-CRM+alum+INI-4001 (*n* = 16) as self-administration of the fentanyl training dose (2.5 µg/kg/infusion) continued. **a** Absolute infusion data (mixed-model ANOVA with Geisser-Greenhouse correction followed by Holm-Sidak multiple comparison tests, statistical symbols: different from baseline, ***p < 0.01; F_1_-CRM+alum+INI-4001 vs CRM, ^#^*p* < 0.05). **b** Data from **a** expressed as a percentage of baseline (mixed-model or one-way ANOVA with Geisser–Greenhouse correction followed by Holm–Sidak multiple comparison tests, statistical symbols: different from CRM, **p < 0.01, ***p < 0.001; F_1_-CRM+alum vs F_1_-CRM+alum+INI-4001, ^#^*p* < 0.05). **c**, **d** Mean (±SEM) fentanyl infusions during the last three sessions at each fentanyl unit dose and saline (0 µg/kg) in rats given CRM (*n* = 11), F_1_-CRM+alum (*n* = 13), or F_1_-CRM+alum+INI-4001 (*n* = 12) expressed as absolute values (**c**) or as a percentage of baseline (i.e., pre-vaccination, **d**). Data analyzed by mixed-model ANOVA with Geisser-Greenhouse correction followed by Holm–Sidak multiple comparison tests. Statistical symbols: different from CRM, *P < 0.05, **P < 0.01, ***P < 0.001. Different from F_1_-CRM-alum, ^#^*P* < 0.05, ^##^*P* < 0.01.

Previous studies reported that vaccines are effective in attenuating the reinforcing properties of fentanyl in operant self-administration models involving lever pressing in rats and non-human primates^20,23,44^. Here, we investigated whether addition of INI-4001 to the lead F_1_-CRM conjugate vaccine increased its efficacy in attenuating fentanyl’s reinforcing effects in rats. Using a fentanyl intravenous self-administration (FSA) model, the mean number of infusions was assessed in rats self-administering fentanyl (2.5 µg/kg/infusion, i.v.) under a fixed ratio (FR) 3 schedule at two weeks following each vaccination during the initial course of four vaccine injections. Data were analyzed as absolute values (Fig. 4a) and as a percentage of baseline (Fig. 4b) to better represent the within-subject effects of vaccination. During this phase, there was a main effect of injection number (mixed-model ANOVA with Geisser–Greenhouse correction, *F*_(3, 110)_ = 27.85, *p* < 0.0001) and an injection number x vaccine group interaction (mixed-model ANOVA with Geisser-Greenhouse correction, *F*_(8, 148)_ = 6.73, *p* < 0.0001), but no effect of vaccine group on absolute number of infusions. Multiple comparisons indicated that only the F_1_-CRM+alum+INI-4001 group showed a significant increase in absolute infusions after the fourth injection (Holm–Sidak’s, *t*_(22)_ = 3.01, *p* < 0.05; a), and the increase in this group approached significance after the third injection (Holm–Sidak’s, *t*_(23)_ = 2.35, *p* = 0.08). There was a significant main effect of injection number, group, and an injection number x group interaction (mixed-model ANOVA with Geisser-Greenhouse correction, *F*_(3, 97)_ = 32.4, *p* < 0.0001, *F*_(2, 39)_ = 14.31, *p* < 0.0001, and *F*_(8, 148)_ = 7.54, *p* < 0.0001, respectively) on the number of infusions as a percentage of baseline. Both vaccine groups, but not the control group, exhibited a significant percent increase in mean infusions over the initial course of four injections, but this increase was significantly greater in F_1_-CRM+alum+INI-4001 group than F_1_-CRM+alum group after the third injection (Holm–Sidak’s, *t*_(23)_ = 2.08, *p* < 0.05; b) and approached significance after the fourth injection (Holm-Sidak’s, *t*_(19)_ = 1.97, *p* = 0.06).

The mean number of self-administered fentanyl infusions was then assessed across a range of unit doses, with each dose available for five sessions. Data were examined as absolute values (Fig. 4c) and a percentage of baseline (Fig. 4d). The baseline for calculating data in Fig. 4d is the self-administration rate at the 2.5 µg/kg fentanyl unit dose prior to vaccination. There was a main effect of vaccine group, fentanyl dose, and group × dose interaction (mixed-model ANOVA with Geisser-Greenhouse correction, *F*_(2, 33)_ = 6.07, *p* < 0.01, *F*_(3, 90)_ = 26.63, *p* < 0.0001, and *F*_(12, 183)_ = 9.54, *p* < 0.0001, respectively) on absolute mean infusions. There was also a main effect of vaccine group and fentanyl dose, and a significant vaccine group × fentanyl dose interaction (mixed-model ANOVA with Geisser-Greenhouse correction, *F*_(2, 33)_ = 4.21, *p* < 0.05, *F*_(3, 88)_ = 28.58, *p* < 0.0001, and F_(12, 183)_ = 9.78, *p* < 0.0001, respectively) on the percent change in mean infusions. Overall, the FSA dose-response curve was shifted to the right in the vaccinated groups compared to controls, as indicated by higher infusion rates at the highest fentanyl doses and lower infusion rates at low fentanyl doses. However, this shift was much more apparent in the F_1_-CRM+alum+INI-4001 group than in the F_1_-CRM+alum group. FSA was significantly decreased compared to controls in the F_1_-CRM+alum+INI-4001 group at all doses below 1.0 µg/kg, whereas a decrease was only apparent at the lowest fentanyl dose in the F_1_-CRM+alum group. In addition, although FSA was increased at the two highest fentanyl doses in both vaccinated groups, the increase was greater in F_1_-CRM+alum+INI-4001 rats than F_1_-CRM+alum rats at the 2.5 µg/kg dose (Fig. 4d), consistent with the greater increase in FSA in this group during the initial course of vaccination.

**Fig. 5:**
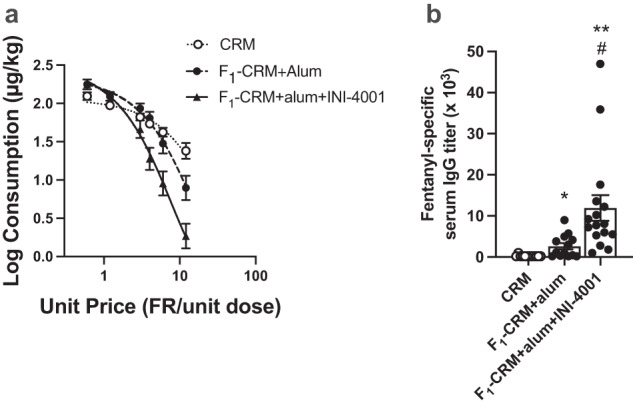
Effect of TLR7/8 adjuvanted anti-fentanyl vaccine on elasticity of demand (α) for fentanyl and antibody titers. **a** Mean (±SEM) fentanyl consumption (mg/kg) as a function unit price in rats given CRM, F_1_-CRM+alum, or F_1_-CRM+alum+INI-4001. Curves are fit to the data using an exponential function (see Methods). Demand curve parameters are presented in Table 2. Data were analyzed by Brown-Forsythe and Welch ANOVA followed by Dunnett’s T3 tests. **b** Mean (±SEM) antibody titers in rats given CRM, F_1_-CRM+alum, or F_1_-CRM+alum+INI-4001 during the FSA study. Samples were acquired 7–14 days after the fourth immunization. Data analyzed by Brown–Forsythe and Welch ANOVA followed by Dunnett’s T3 tests. Statistical symbols: different from CRM, *P < 0.05, **P < 0.01; different from F_1_-CRM+alum, ^#^*P* < 0.05.

To directly examine the effect of vaccination on the reinforcing efficacy of fentanyl, behavioral economic demand curves were compared between groups (Fig. 5a). In this analysis, an exponential curve is fit to the data to describe the relationship between fentanyl consumption and unit price (FR/unit dose). This yields several parameters of demand (Table 2), including the rate of decline in consumption as unit price increases (i.e., elasticity of demand (*α*)), where a faster decline (greater elasticity, higher *α*) indicates lower reinforcing efficacy; the estimated level of consumption at zero price (i.e. intensity of demand (*Q*_0_)); the unit price at which consumption changed from relatively inelastic to relatively elastic (*P*_max_, lower values indicate lower reinforcing efficacy); and the maximum level of responding for fentanyl (*O*_max_, lower values indicate lower reinforcing efficacy). The change in fentanyl consumption in each group with increases in unit price was well described by the exponential demand function (mean *r*^2^ did not differ between groups). Overall, F_1_-CRM+alum+INI-4001 reduced the reinforcing efficacy of fentanyl to a greater degree than F_1_-CRM+alum. Elasticity of demand (*α*) was significantly higher (i.e., reinforcing efficacy was lower) than CRM in the F_1_-CRM+alum+INI-4001 group (Dunnett’s T3, *t*_(20)_ = 2.61, *p* < 0.05), but not the F_1_-CRM+alum group. Consistent with the vaccine effects on maintenance of FSA (Fig. 4), intensity of demand (*Q*_0_), was significantly higher compared to CRM in both the F_1_-CRM+alum group (Dunnett’s T3, *t*_(16)_ = 3.51, *p* < 0.01) and F_1_-CRM+alum+INI-4001 (Dunnett’s T3, *t*_(16)_ = 4.51, *p* < 0.001) groups, but the vaccines did not differ from each other. *P*_max_, was significantly lower than controls in both F_1_-CRM+alum (Dunnett’s T3, *t*_(15)_ = 4.10, *p* < 0.01) and F_1_-CRM+alum+INI-4001 rats (Dunnett’s T3, *t*_(11)_ = 6.48, *p* < 0.001), but this effect was greater in F_1_-CRM+alum+INI-4001 rats (Dunnett’s T3, *t*_(15)_ = 2.93, *p* < 0.05). *O*_max_, was significantly lower than CRM in the F_1_-CRM+alum+INI-4001 group (Dunnett’s T3, *t*_(20)_ = 2.61, *p* < 0.05), but not the F_1_-CRM+alum group.

**Table 2:** Mean (±SEM) exponential demand curve parameter estimates from individual rat curve fits (group fits in Fig. 6).

Parameter	CRM	F_1_-CRM+alum	F_1_-CRM+alum+INI-4001
*α*	0.000170 (0.000028)	0.000218 (0.000046)	0.000291 (0.000043)*
*Q*_0_	118.1 (12.3)	250.8 (51.7)**	301.9 (52.5)**
*P*_max_	8.65 (0.99)	3.90 (0.60)**	1.95 (0.31)**^, #^
*O*_max_	329.1 (36.5)	290.6 (41.9)	203.5 (31.2)*
*R*^2^	0.88 (0.04)	0.94 (0.02)	0.93 (0.02)

Range constant *k* = 3.63. Different from CRM *p < 0.05, **p < 0.01. Different from *F*_1_-CRM+alum, ^#^*p* < 0.05.

Bold entries denote statistically significant values.

Differences in FSA measures between groups were consistent with differences in mean fentanyl-specific antibody titers between groups measured following the 4th immunization (Fig. 5b). Titers were significantly higher than control in F_1_-CRM+alum and F_1_-CRM+alum+INI-4001 rats (Dunnett’s T3, *t*_(12)_ = 3.1, *p* < 0.05 and *t*_(15)_ = 3.75, *p* < 0.01, respectively), and titers in the F_1_-CRM+alum+INI-4001 rats were significantly higher than those in F_1_-CRM+alum rats (Dunnett’s T3, *t*_(17)_ = 2.91, *p* < 0.05).

### INI-4001 increases vaccine efficacy in a porcine model of fentanyl overdose toxicity

**Fig. 6:**
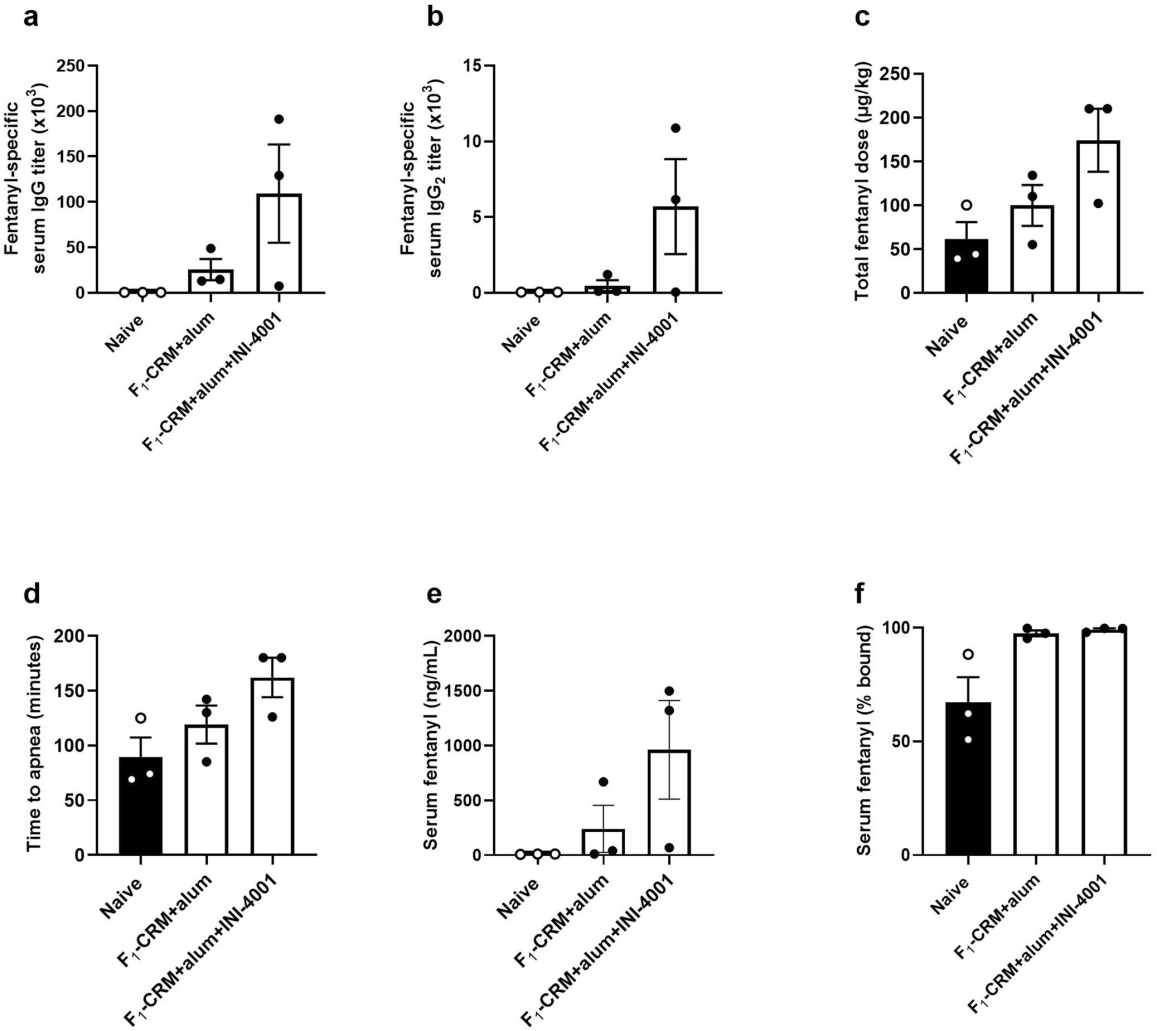
INI-4001 increases efficacy of an anti-fentanyl vaccine in a porcine model of fentanyl-induced respiratory depression. Pigs (*n* = 3/group) were immunized on days 0, 21, and 42. Blood was collected on day 49 ± 3 to measure **a** fentanyl-specific serum total IgG titers and **b** IgG_2_ subclass titers via ELISA. Starting on day 49 ± 3, pigs were challenged with a continuous IV infusion of fentanyl that increased over time until two minutes of continuous apnea was reached. **c** Total fentanyl dose received before apnea was achieved. **d** Time until apnea was achieved. **e** Total serum fentanyl concentration at time of apnea, measured via LC-MS. **f** Serum fentanyl expressed as percent (%) protein-bound. Data are mean ± SEM.

While rodent models are more common and convenient for vaccination and drug-challenge studies, both mice and rats have differentially functioning TLR8 signaling compared to humans^41,45^, making it necessary to test this lead fentanyl vaccine formulation in a model that more closely simulates the human immune system. The immune system of Hanford mini-pigs is 80% similar to humans (compared to 10% similarity between humans and rodents)^46^, and their immune cells express both TLR7 and TLR8 which have a similar function as in humans^41,46^. Therefore, we developed a porcine model of vaccination and opioid-induced respiratory depression to test the effects of INI-4001 on anti-fentanyl vaccination. In a pilot study, pigs were immunized with F_1_-CRM adsorbed on alum or F_1_-CRM and INI-4001 adsorbed on alum on days 0, 21, and 42. Serum was collected 7 days after each vaccination for titer determination. On day 49, mini-pigs receiving INI-4001 adjuvanted vaccine had trending, higher titers of total fentanyl-specific IgG (Fig. 6a) and fentanyl-specific IgG_2_ (Fig. 6b)—an antibody subclass that may be similar to IgG_2a_ in rodents^47,48^. After immunization, pigs were anesthetized with isoflurane and challenged with a continuous IV infusion of fentanyl until two minutes of continuous apnea was reached. Serum samples were collected at multiple timepoints to measure fentanyl pharmacokinetics. Total fentanyl dose (Fig. 6c), time to apnea (Fig. 6d), and serum fentanyl concentration at time of apnea (Fig. 6e) were measured as parameters for vaccine efficacy. Groups immunized with F_1_-CRM+alum+INI-4001 had trended towards higher tolerated fentanyl dose, time to apnea, and serum fentanyl concentrations, indicating that INI-4001 may be increasing the overall efficacy of the fentanyl vaccine (other measured parameters can be found in Supplementary Fig. 2). Serum collected at time the of apnea (or at the final challenge timepoint, if the pig never became apneic) was also analyzed for the ratio of protein-bound fentanyl compared to free fentanyl (Fig. 6f). The mean ± SEM protein-bound serum fentanyl was 67.2% ± 11.1% in naïve pigs at time of apnea, which is generally consistent with literature describing fentanyl plasma protein binding in humans^49^. Conversely, the F_1_-CRM+alum and F_1_-CRM+alum+INI-4001 groups had 97.6% ± 1.3% and 99.2% ± 0.6% protein-bound fentanyl, respectively, indicating that the increase in serum fentanyl seen after drug challenge represents almost entirely antibody-bound drug.

**Fig. 7:**
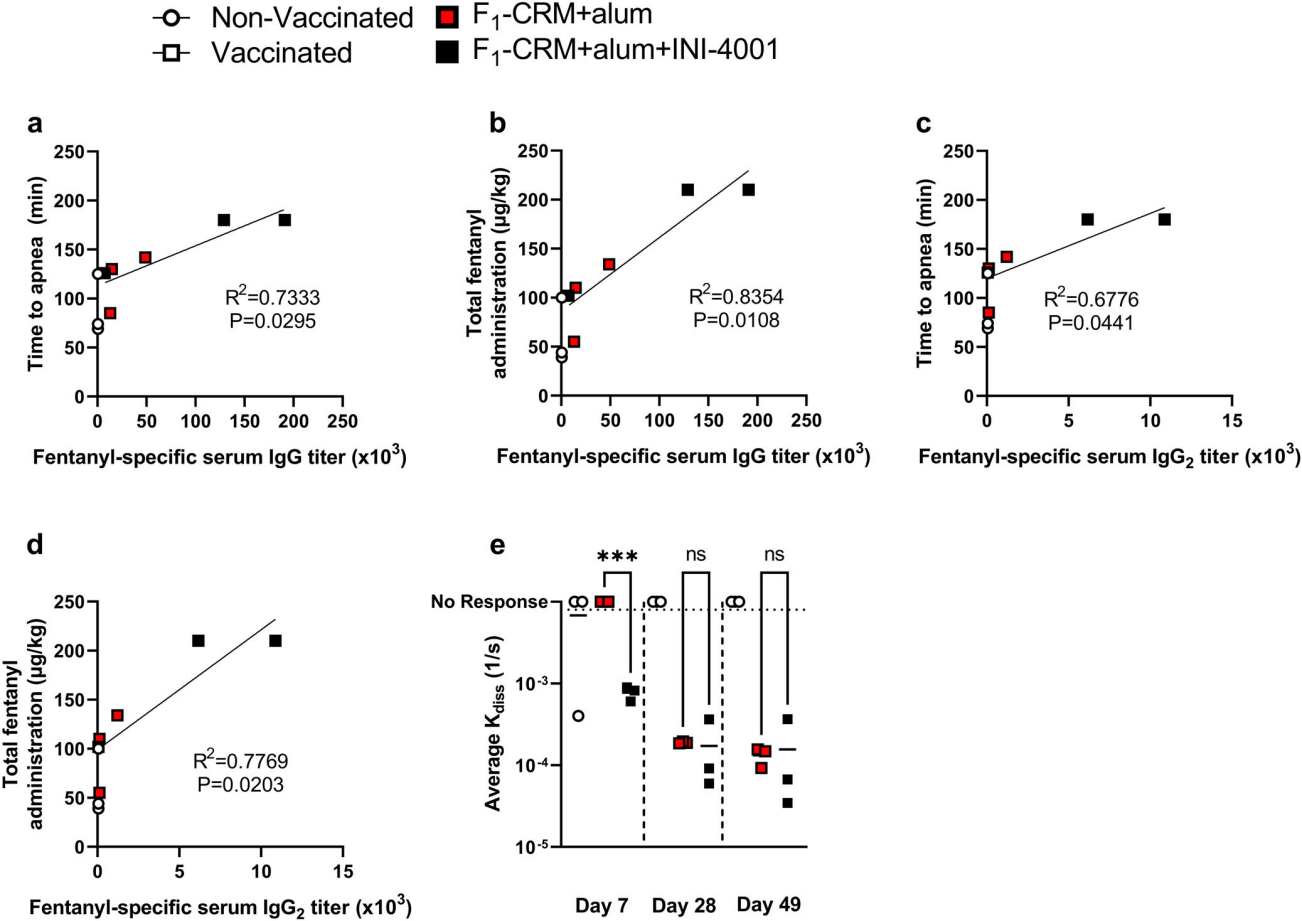
Porcine serum antibody titers are associated with increased vaccine efficacy. Pig total serum IgG titers were plotted against **a** time to apnea and **b** total fentanyl administration at time of apnea. Pig total serum IgG_2_ subclass titers were plotted against **c** time to apnea and **d** total fentanyl administration at time of apnea. **e** Polyclonal antibody avidity was measured in all groups at three timepoints using biolayer interferometry. Statistical analysis was performed via Pearson correlation after determining data normality using D’Agostino–Pearson’s test (**a**–**d**), or one-way ANOVA followed by Tukey’s multiple comparison’s post hoc test (**e**). Statistical symbols: ***P < 0.001.

The serum fentanyl-specific total IgG and IgG_2_ antibodies were further analyzed by plotting them against parameters of vaccine efficacy. Higher titers of both total IgG and IgG_2_ were significantly associated with greater vaccine efficacy, including a longer time to apnea and greater total fentanyl administration (Fig. 7a–d). Additionally, the avidity of these antibodies was measured via biolayer interferometry at each timepoint after vaccination. The addition of INI-4001 increased early antibody avidity compared to F_1_-CRM+alum, but the *K*_diss_ between groups was similar at both day 28 and day 49 (Fig. 7e). Overall, these data suggest that mini-pigs can be used as a model for fentanyl-induced respiratory depression and vaccination against opioids, and that INI-4001 increases the efficacy of a lead anti-fentanyl vaccine in this model.

## Discussion

Active immunization with vaccines targeting fentanyl and its analogs is a potentially safe, long-lasting, and prophylactic treatment to combat OUD and overdose in addition to the existing treatment repertoire of pharmacotherapies. While anti-opioid vaccines have shown significant preclinical efficacy at reducing drug self-administration and preventing symptoms associated with fatal opioid overdose such as bradycardia and respiratory depression^10–23^, clinical evaluation of these vaccines has only recently been initiated with one Phase Ia/Ib clinical trial currently being conducted (NCT04458545). Lessons learned from clinical trials of other anti-drug conjugate vaccines reveal that high concentrations of high-affinity antibodies are needed to achieve significant efficacy^24,25^. As such, efforts have focused on increasing the immune response to opioid-specific vaccines through optimization of conjugation chemistry, linker length, hapten design, formulation with adjuvant, delivery platforms, immunization schedule, and identification of biomarkers associated with better antibody responses^10,12,18,21,26,27,29,30,33,50–57^.

Aluminum hydroxide (alum) is the most widely used adjuvant in FDA-approved vaccines^28,58^. While it has been used for decades to improve immune responses to many vaccines^58^, its exact mechanism of action is unclear^28^. Alum has been used in most studies involving anti-opioid vaccines^9^ due to the ease of translation into clinical settings because of its known safety and efficacy profile. However, alum drives a Th_2_ skewed response and elicits the production of IgG_1_ antibodies in mice^28^. Published literature suggests that optimal anti-opioid immune response in mice may consist of a balanced Th_1_/Th_2_ response and production of both IgG_1_ and IgG_2a_ antibodies^26,27^. While alternative adjuvants have been tested with anti-opioid vaccines^12,21,26,27,29–33^, many of these adjuvants are not well tolerated, exhibited minimal efficacy, or have limited availability due to cost or patent considerations. Therefore, we initially tested the addition of synthetic TLR4 (INI-2002) and TLR7/8 (INI-4001) adjuvants adsorbed on alum to produce a safe and balanced Th_1_/Th_2_ response with the goal of increasing vaccine immunogenicity and efficacy. We found that the TLR7/8 agonist increased total IgG and IgG_2a_ subclass titers, which led to increased vaccine efficacy after fentanyl challenge in mice (data published in concurrent dual submission to NPJ Vaccines). Addition of TLR7/8 achieved efficacy despite the antigen dose being reduced over 10× compared to previous formulations containing alum alone^20^ (data published in concurrent dual submission to NPJ Vaccines), which may support use of this adjuvant in dosing-sparing formulations. Both adjuvanted vaccines were well tolerated across the dose ranges evaluated. To extend these findings, the present study tested the same adjuvanted vaccines in rats and found that vaccines adjuvanted with INI-4001 (TLR7/8 agonist), but not INI-2002 (TLR4 agonist), significantly increased rat serum IgG titers, which increased serum fentanyl concentration and decreased brain fentanyl concentration after drug challenge. This is consistent with our studies in mice (concurrent dual submission to NPJ Vaccines) and previous studies indicating that TLR4 agonists, such as MPLA, do not improve efficacy of oxycodone conjugate vaccines when used as vaccine adjuvants^29^.

Advancing INI-4001 as our candidate adjuvant for fentanyl vaccine development, we further tested its ability to protect against higher-dose fentanyl challenges simulating overdose situations by assessing resistance to fatal levels of respiratory depression and determining dose-response curves after repeated doses of fentanyl. The absolute EC_50_ values determined from these dose-response curves would likely be inaccurate since the study was performed using a cumulative dosing paradigm. Since doses were scheduled every 15 minutes, free fentanyl was being actively metabolized throughout the duration of the study, leading to the reported cumulative dose likely being higher than the active physiological dose. Nonetheless, comparing the magnitude of shift in the midpoint of the dose-response curve between groups is informative. We found that the addition of INI-4001 to F_1_-CRM+alum shifted fentanyl’s dose-response curve almost 3-fold compared to formulations containing alum alone and elicited greater protection against respiratory depression after drug challenge.

The increase in antibody titers and protection against fentanyl found when animals were immunized with INI-4001 adjuvanted F_1_-CRM does not appear to be at the expense of specificity towards the target drug, as the present study found that there were no significant changes in cross-reactivity between F_1_-CRM formulations containing INI-4001 adsorbed on alum or with just alum alone. Some cross-protection against additional fentanyl analogs has been previously reported^20,34^, and the current study suggests that this cross-protection will not be affected by the inclusion of INI-4001 as a vaccine adjuvant. Importantly, there was negligible cross-reactivity to medications for OUD management such as methadone, buprenorphine, and naltrexone, or medications for overdose reversal such as naloxone. This is critical for the future translation of fentanyl vaccines into clinical settings, as these vaccines will likely be utilized in combination with other OUD or overdose medications.

In a previous FSA study, fentanyl intake decreased in rats vaccinated with 60 μg F_1_-CRM and 90 μg alum^20^. In the current FSA study, fentanyl intake increased in F_1_-CRM+alum and F_1_-CRM+alum+INI-4001 vaccinated rats during maintenance at the 2.5 µg/kg dose relative to pre-vaccination intake and increased intake at the 5.0 µg/kg dose during the fentanyl dose-response determination. In contrast, intake was decreased at lower unit doses during the dose-response determination, and the reduction was greater in F_1_-CRM+alum+INI-4001 compared to F_1_-CRM+alum vaccinated rats. This rightward shift in the unit dose-response curve indicates that the F_1_-CRM immunogen reduced the potency of fentanyl (i.e., effectively lowering the fentanyl dose), and that the addition of the TLR7/8 agonist INI-4001 significantly enhanced this effect. We have reported similar shifts in potency produced by analogous oxycodone and heroin vaccines in oxycodone and heroin self-administration models^15,59^, and others have shown a similar reduction in potency in fentanyl self-administration models^44,60^. In addition, behavioral economic demand analysis showed F_1_-CRM+alum+INI-4001 was more effective at increasing the elasticity of demand (i.e., increasing α and decreasing *P*_max_) and decreasing maximal response for fentanyl, indicating a greater decrease in the reinforcing efficacy of fentanyl and rats’ motivation to consume it. However, it is important to note that our demand curve analysis involved manipulation of unit price by changing the unit dose rather than the more conventional approach of changing the FR (response cost). It is possible that demand curve parameters and vaccine effects under changing FR requirements may differ somewhat from the present study. Our approach nonetheless provides a valid and precise method for comparing fentanyl consumption between groups. To our knowledge, this is the first direct demonstration of a TLR agonist adjuvant enhancing the efficacy of any drug abuse vaccine in attenuating the reinforcing effects of a drug. These data suggest that F_1_-CRM+alum+INI-4001 may be effective at facilitating abstinence from fentanyl in humans by blocking its reinforcing effects. These results also suggest that adjuvants targeting TLR7/8 receptors enhance this effect, which may generalize to vaccines against other drugs of abuse.

The increase in fentanyl intake observed at higher unit doses in vaccinated rats has important clinical implications. The FSA maintenance model in the present study was intended to model vaccination in early clinical trials, where participants may be users of fentanyl who continue using the drug while they are being vaccinated. As such, the increase in FSA during vaccination in the present study suggests that a compensatory increase in fentanyl use might also occur in humans during immunization. Some of these concerns may be mitigated by the fact that no compensatory increases were found in previous FSA studies of F_1_-CRM+alum^20^. It is possible that the higher dose of F_1_-CRM and alum and the lower fentanyl training dose (1.0 μg/kg/infusion) may have elicited a different ratio of available antibody binding sites over moles of circulating fentanyl molecules. Additionally, it has been shown that precipitating withdrawal with an opioid antagonist can motivate increases in opioid self-administration in rats^61^. Indeed, withdrawal has long been thought to play a key role in motivating drug use in humans^62–64^. This raises the question of whether the increase in FSA observed in the present study was precipitated by a vaccine-induced withdrawal state. To the extent that it was, this would suggest that adjunct treatments (e.g., methadone, buprenorphine) might be needed to minimize this potential side effect of vaccination and avoid treatment noncompliance in clinical trials (i.e., not showing up for vaccinations). However, the gradual rise in antibody levels during vaccination differs from the relatively rapid blockade of opioid receptors by an opioid antagonist and may therefore be too slow to precipitate withdrawal. Accordingly, precipitated withdrawal has not been reported in preclinical studies with other addiction vaccines or anti-drug monoclonal antibodies^65,66^. Moreover, the level of access to fentanyl (only 2 hrs/day, 5 days/week) may not have been sufficient to induce dependence on fentanyl^61^. Nonetheless, ongoing and future preclinical and clinical research will focus on assessing the extent to which opioid vaccines might precipitate withdrawal and how to optimize the combination of immunotherapies and pharmacotherapies to maximize outcomes.

Potentially clinically relevant differences in TLR7/8 expression and functionality have been described between rodents and humans. TLR8 is expressed and functional in human monocytes and conventional dendritic cells (cDCs) with little to no expression in plasmacytoid dendritic cells (pDCs) and B cells^40,41,67^. TLR7 is expressed by pDCs and B cells in humans, and in contrast by pDCs, B cells, monocytes, and cDCs in mice^68,69^. While both mouse and human TLR7 respond to the same structures and compounds, compounds specific to human TLR8 instead activate mouse TLR7. While mouse TLR8 was initially thought to be non-functional, more recent studies show that it responds to different structures and compounds compared to human TLR8^40–42,67,70^. Pigs have been used as an alternative species for the evaluation of TLR7/8 ligands based on literature reports demonstrating more human-like responsiveness to TLR7 and TLR8 ligands^42^. Thus, the lead vaccine formulation containing INI-4001 was tested in Hanford miniature pigs, which provided a relevant animal model to recapitulate human TLR7/8 vaccine efficacy and tolerability. To demonstrate vaccine efficacy in this species, we developed a model of respiratory depression after fentanyl challenge. While there have been reports of porcine models of pharmacokinetics of opioids such as morphine^71^, and models of brain tissue hypoxia after opioid overdose^72^, to our knowledge this is the first report of a porcine model of fentanyl-induced respiratory depression and overdose after anti-fentanyl vaccination. After immunization of Hanford miniature pigs with F_1_-CRM adsorbed on alum or F_1_-CRM and INI-4001 adsorbed on alum in a pilot study, we saw trending increases in total IgG and IgG_2_ subclass titers, similar to findings in mice (data published in concurrent dual submission to NPJ Vaccines) and rats. No vaccine-related adverse events or injection site reactogenicity was noted in any vaccinated pigs. Furthermore, we found that pigs immunized with INI-4001 adjuvanted F_1_-CRM tolerated a higher fentanyl dose and were more resistant to becoming apneic compared to naïve or F_1_-CRM+alum vaccinated pigs. Indeed, two out of three pigs in the INI-4001 adjuvanted group did not become apneic, even at the highest dose of fentanyl tested. Similarly, we found that there was a trend toward higher levels of serum fentanyl at the time of apnea, of which almost all is protein-bound, suggesting that more fentanyl was retained in the serum and not crossing through the blood-brain barrier. This pilot study had a small sample size (*n* = 3/group) and therefore was not powered for accurate statistical analysis; however, future studies will investigate this model with larger sample sizes to confirm the efficacy and safety of the candidate F_1_-CRM+alum+INI-4001 vaccine in this species.

Finally, despite a small sample size, significant correlations were found between opioid-specific antibodies and parameters of vaccine efficacy. No differences in antibody avidity were found between groups, except at the earliest timepoint. This result suggests that efficacy in prevention of fentanyl-induced overdose is primarily dependent on high antibody titers, which is consistent with previous literature in mice, rats, and human clinical trials of other conjugate vaccines^10,16,24,25,27,32,53^. Additionally, there was a significant early increase in avidity in INI-4001 adjuvanted groups despite almost indetectable titers of serum IgG at the same timepoint (Supplementary Fig. 3). This suggests that there may be a more robust early IgM response after INI-4001 administration, which may lead to earlier onset of protection after vaccination compared to a vaccine adjuvanted with alum alone. Studies investigating vaccine-induced immune responses and efficacy against the target drug over multiple early timepoints will be of interest for future clinical evaluation to determine onset of efficacy in human patients.

Overall, as clinical testing of anti-opioid vaccines begins, it is imperative that vaccines are optimized for safety and produce high anti-opioid antibody titers to ensure clinical efficacy. The addition of the synthetic TLR7/8 agonist INI-4001 to our lead anti-fentanyl vaccine significantly increased antigen-specific antibody titers, which led to increases in vaccine efficacy after drug challenge in multiple animal species, drug doses, and administration paradigms. This supports the translation of INI-4001 as an adjuvant for clinical studies of F_1_-CRM, other anti-opioid conjugate vaccines, and potentially vaccines directed against other drug targets.

## Methods

### Hapten synthesis, conjugation, and vaccine formulation

The fentanyl-based hapten containing a tetraglycine linker (F_1_ or F) was synthesized as previously described^19,20^. Briefly, piperidone hydrochloride was alkylated with 2-(N-Boc-aminoethyl) bromide using potassium carbonate in acetonitrile to afford the N-substituted amino ethylpiperidine intermediate. Reductive amination of the ketone with aniline using sodium cyanoborohydride in the presence of an equimolar amount of acetic acid provided the 4-aminophenyl-piperidine precursor. The 4-aminophenyl piperidine precursor was acylated using propionyl chloride in the presence of Hunig’s base [N,N-diisopropylethylamine {DIPEA)]. Acid-mediated N-Boc terminal group deprotection followed by acylation with glutaric anhydride in the presence of pyridine yielded the carboxylic acid precursor. The linker (Gly)_4_-OtBu was then attached using 2- (1H-benzotriazol-1-yl)-1,1,3,3-tetramethyluronium hexafluorophosphate (HBTU) and DIPEA as coupling agents. Finally, the tert-butyl ester was hydrolyzed using 20% trifluoracetic acid in dichloromethane to provide hapten. The F_1_ hapten was then conjugated to either GMP grade diphtheria cross-reactive material (Pfenex, San Diego, CA or Fina Biosolutions, Rockville MD) or bovine serum albumin (BSA) using carbodiimide chemistry as previously described^19,20^. Briefly, haptens were first dissolved at a concentration of 5.2 nM in 0.1 M MES buffer pH 5.0 in the presence of 10% DMSO (w/v), and then haptens were activated by carbodiimide (EDAC, Sigma-Aldrich, St. Louis, MO) at a final concentration of 208 nM. The conjugation reaction was incubated at room temperature for 10 minutes before the carrier protein was added at a final concentration of 2.8 mg/ml. Reactions were then stirred for 3 hours at room temperature. The resulting conjugates were purified by ultrafiltration using Amicon filters to replace MES buffer with phosphate-buffered saline (PBS) 0.1 M pH 7.2. For CRM conjugates, 250 mM sucrose was added in both conjugation and storage buffers for stability. For competitive binding ELISA, a structurally-related fentanyl-based hapten (F_3_) conjugated to BSA was used as a coating antigen, the synthesis of which has been previously described^20^. Briefly, (2*E*)-3-(4-{2-[4-(*N*-Phenylpropanamido)piperidin-1-yl]ethyl}-phenyl)prop-2-enoic acid was added to a solution of 1-ethyl-3-(3-dimethylaminopropyl)carbodiimide-HCl and *N*-hydroxysuccinimide in CH_2_Cl_2,_ for 18 h at room temperature with stirring. H_2_O and EtOc were added to the reaction mixture by stirring for 10 min. The organic layer was then extracted with EtOAc using a separatory funnel, washed with brine, and dried with anhydrous MgSO_4_. The organic solvent was removed under reduced pressure to afford F_3_. The F_3_ was conjugated to BSA as described above. The unconjugated carrier protein or the conjugate vaccines were adsorbed on aluminum adjuvant (Alhydrogel ’85’, 2%, Brenntag Biosector, Denmark) and mixed with TLR agonists as described in each experimental section.

### Adjuvants

#### INI-4001 synthesis

All dry reagents were dried from anhydrous pyridine or toluene and left under high vacuum for 18 hours. All glassware was heat dried and purged with a dry, inert gas. The first two steps were done under argon. To a solution of PEG3 glycerol (1.16 grams, 1.65 mmol) and 2-cyanoethyltetraisopropyl-phosphordiamidite (523 µL, 1.65 mmol) in 20 mL anhydrous methylene chloride was added 1H-tetrazole (115 mg, 1.65 mmol) in 4 portions over 20 minutes. After stirring at room temperature for one hour, the reaction was cooled on an ice bath. To the cooled solution was added UM-3002 (500 mg, 1.20 mmol) and imidazolium triflate (525 mg, 2.41 mmol). After 10 minutes the reaction was removed from the ice bath and stirred at room temperature for one hour. Tert-butyl peroxide (438 µL, 5.5 M in nonane) was added and the reaction stirred at room temperature for 30 minutes. The reaction was quenched with saturated sodium thiosulfate and the organic layer was dried over sodium sulfate and condensed. The crude material was dissolved in 20 mL methylene chloride and DBU (1.80 mL, 12.1 mmol) was added. After 15 minutes, the solution was quenched with 0.1 N HCl and the organic layer dried over sodium sulfate. Purification on silica ([acetonitrile/methanol 1:1]/chloroform) afforded INI-4001 (680 mg, 50%) as an off-white solid. 1H NMR (400 Hz) δ 5.19-5.21 (m, 1H); 5.06-5.10 (m, 1H); 4.34 (dd, J = 11.8 Hz, 1H); 4.12-4.19 (m, 3H); 4.04 (dt, 6.6 Hz, 2H); 3.58-3.77 (m, 14H); 3.26 (bs, 2H); 2.77(bs, 1H); 2.25–2.34 (m, 5H); 1.74–1.95 (m, 4H); 1.72–1.74 (m, 1H); 1.52–1.61 (m, 5H); 1.37–1.47 (m, 3H); 1.25–1.31 (m, 50H); 0.93 (t, 7.2 Hz, 3H); 0.88 (t, 6.8 Hz, 6H). Anal. Calcd for C59H109N6O13P: C (62.08), H (9.63), N (7.36). Found: C (61.79), H (9.71), N (7.24).

#### INI-2002 synthesis

(1) A solution of 1,3,4,6-tetra-*O*-acetyl-2-amino-2-deoxy-β-d-glucopyranose hydrochloride (76.47 g, 0.23 mol) in methylene chloride (350 mL) and H_2_O (350 mL) was treated with sodium bicarbonate (149.94 g, 1.79 mol) added in portions slowly. Benzyl chloroformate (79.17 g, 0.46 mol) was added in portions to control gas evolution and the reaction was stirred vigorously for 2.5 hours. The layers were separated and the aqueous layer was extracted with methylene chloride (100 mL). The combined organic layers were washed with saturated aqueous sodium chloride, dried over anhydrous sodium sulfate, filtered, and concentrated to approximately 100 mL. Methyl-*t*-butyl ether (200 mL) was added and the resulting mixture was stirred and cooled to 0 °C the precipitate was collected by filtration, washed with cold methyl-*t*-butyl ether, and dried in a vacuum oven to give 88.89 g (81%) of 1,3,4,6-tetra-*O*-acetyl-2-(benzyloxycarbonylamino) 2-deoxy-β-D-glucopyranoside. (2) A solution of 1,3,4,6-tetra-*O*-acetyl-2-(benzyloxycarbonylamino) 2-deoxy-β-d-glucopyranoside (10 g, 20.8 mmol) and benzyl *N*-(2-hydroxyethyl)carbamate (4.48 g, 22.9 mmol) in anhydrous methylene chloride (80 mL), cooled to −15 °C, was treated dropwise with trimethylsilyl triflate (0.37 mL, 2.08 mmol). The reaction mixture was allowed to warm to room temperature over 5.5 hours. The reaction was quenched with saturated aqueous sodium bicarbonate (40 mL) and the layers were separated. The aqueous layer was extracted with methylene chloride (2 × 20 mL) and the combined organic layers were dried over anhydrous sodium sulfate, filtered, and concentrated in vacuo. The crude product obtained was crystallized from methylene chloride/heptane to give 10.4 g (81%) of 2-(benzyloxycarbonylamino)ethyl 3,4,6-tri-*O*-acetyl-2-benzyloxycarbonylamino-2-deoxy-β-D-glucopyranoside as a white solid. (3) A solution of 2-(benzyloxycarbonylamino)ethyl 3,4,6-tri-*O*-acetyl-2-benzyloxycarbonylamino-2-deoxy-β-D-glucopyranoside (10 g, 16.3 mmol) in methanol (160 mL) was treated with ammonium hydroxide (20 equivalents) for 2 hours at room temperature. The reaction mixture was concentrated and dried under high vacuum overnight to give 8 g (100%) of 2-(benzyloxycarbonylamino)ethyl 2-benzyloxycarbonylamino-2-deoxy-β-D-glucopyranoside as a white solid, which was used without further purification. (4) A solution of 2-(benzyloxycarbonylamino)ethyl 2-benzyloxycarbonylamino-2-deoxy-β-D-glucopyranoside (8 g, 16.3 mmol) in acetonitrile (180 mL) was treated with benzaldehyde dimethyl acetal (4.9 mL, 32.6 mmol) and camphorsulfonic acid (1.9 g, 8.2 mmol). The reaction was stirred for 3 hours, neutralized with saturated aqueous sodium bicarbonate, filtered, and concentrated in vacuo. The crude product was crystallized from ethyl acetate/heptane to give 7.1 g (75%) of 2-(benzyloxycarbonylamino)ethyl 4,6-*O*-benzylidene-2-deoxy-2-benzyloxycarbonylamino-2-deoxy-β-D-glucopyranoside as a white solid. (5) A solution of 2-(benzyloxycarbonylamino)ethyl 4,6-*O*-benzylidene-2-deoxy-2-benzyloxycarbonylamino-2-deoxy-β-D-glucopyranoside (1.5 g, 2.59 mmol) in anhydrous tetrahydrofuran (40 mL) was treated with triethylamine (0.54 mL, 3.89 mmol) and triphenylphosphine (1.09 g, 4.14 mmol). The reaction mixture was cooled to 0 °C and diisopropyl azodicarboxylate (0.82 mL, 4.14 mmol) was added. After 45 minutes at 0 °C, diphenylphosphoryl azide (0.89 mL, 4.14 mmol) was added. The reaction was allowed to gradually warm up to room temperature and stirring continued for 18 hours. The reaction mixture was concentrated in vacuo and the residue chromatographed on silica gel (gradient elution, 20→70% ethyl acetate/heptane) affording 1.16 g (74%) of 2-(benzyloxycarbonylamino)ethyl 3-azido-4,6-*O*-benzylidene-2-benzyloxycarbonylamino-2,3-dideoxy-β-D-allopyranoside as a white solid. (6) A solution of 2-(benzyloxycarbonylamino)ethyl 3-azido-4,6-*O*-benzylidene-2-benzyloxycarbonylamino-2,3-dideoxy-β-D-allopyranoside (2.95 g, 4.89 mmol) in anhydrous tetrahydrofuran (100 mL) was treated with a solution of 0.1 N sodium hydroxide (9.8 mL, 0.98 mmol) and a solution of 1.0 M of trimethylphosphine in tetrahydrofuran (7.8 mL, 7.82 mmol). The reaction stirred at room temperature for 18 hours. The reaction mixture was concentrated in vacuo. The residue was chromatographed on silica gel (gradient elution, 30→100% ethyl acetate/heptane then 0→10% methanol/chloroform) affording 2.37 g (84%) of 2-(benzyloxycarbonylamino)ethyl 3-amino-4,6-*O*-benzylidene-2-benzyloxycarbonylamino-2,3-dideoxy-β-D-allopyranoside as a white solid. (7) A solution of 2-(benzyloxycarbonylamino)ethyl 3-amino-4,6-*O*-benzylidene-2-benzyloxycarbonylamino-2,3-dideoxy-β-D-allopyranoside (0.5 g, 0.87 mmol) in anhydrous methylene chloride (10 mL) was acylated with (*R*)-3-decanoyloxytetradecanoic acid (414 mg, 1.04 mmol) and 1-(3-dimethylaminopropyl)-3-ethylcarbodiimide methiodide (310 mg, 1.04 mmol) at room temperature for 2 hours. The reaction mixture was quenched with saturated aqueous sodium bicarbonate (5 mL) and the layers separated. The aqueous layer was extracted with chloroform (2 × 5 mL) and the combined organic layers were washed with water (5 mL), dried over anhydrous sodium sulfate and concentrated in vacuo. Chromatography on silica gel (gradient elution, 10→ 60% ethyl acetate/heptane) afforded 748 mg (90%) of 2-(benzyloxycarbonylamino)ethyl 4,6-*O*-benzylidene-2-benzyloxycarbonylamino-3-[(*R*)-3-decanoyloxytetradecanoylamino]-2,3-dideoxy-β-d-allopyranoside as a colorless oil. (8) A solution of 2-(benzyloxycarbonylamino)ethyl 4,6-*O*-benzylidene-2-benzyloxycarbonylamino-3-[(*R*)-3-decanoyloxytetradecanoylamino]-2,3-dideoxy-β-D-allopyranoside (745 mg, 0.78 mmol) in anhydrous tetrahydrofuran (20 mL) was hydrogenated with 10% palladium on carbon (220 mg) using a Parr hydrogenator at room temperature and 50 psig for 24 hours. The reaction mixture was filtered through Celite and the filtrate concentrated in vacuo. The resulting oil dissolved in methylene chloride (10 mL) was acylated with (*R*)-3-decanoyloxytetradecanoic acid (680 mg, 1.71 mmol) and 1-(3-dimethylaminopropyl)-3-ethylcarbodiimide methiodide (510 mg, 1.71 mmol) at room temperature for 2 hours. The reaction mixture was quenched with saturated aqueous sodium bicarbonate (10 mL) and the layers separated. The aqueous layer was extracted with methylene chloride (2 x 10 mL) and the combined organic layers washed with water (10 mL), dried over anhydrous sodium sulfate and concentrated in vacuo. Chromatography on silica gel (gradient elution, 20→80% ethyl acetate/heptane) afforded 732 mg (65%) of 2-[(*R*)-3-decanoyloxytetradecanoylamino]ethyl 4,6-*O*-benzylidene-2,3-di-[(*R*)-3-decanoyloxytetradecanoylamino]-2,3-dideoxy-β-D-allopyranoside as a glassy solid. (9) A solution of 2-[(*R*)-3-decanoyloxytetradecanoylamino]ethyl 4,6-*O*-benzylidene-2,3-di-[(*R*)-3-decanoyloxytetradecanoylamino]-2,3-dideoxy-β-D-allopyranoside (400 mg, 0.282 mmol) in anhydrous methylene chloride (20 mL) cooled to 0 °C was treated with sodium cyanoborohydride (42 mg, 0.655 mmol) followed by the addition of trifluoroacetic acid (0.06 mL, 0.786 mmol). The reaction mixture gradually warmed up to room temperature and continued to stir for 3 hours. The reaction was quenched with methanol (2 mL), concentrated in vacuo then reconstituted in methylene chloride and washed with a saturated solution of sodium bicarbonate. The layers separated and the aqueous layer was extracted with methylene chloride (2 x 10 mL) and the combined organic layers dried over anhydrous sodium sulfate and concentrated in vacuo. Chromatography on silica gel (gradient elution, 10→ 95% ethyl acetate/heptane) afforded 380 mg (93%) of 2-[(*R*)-3-decanoyloxytetradecanoylamino]ethyl 6-*O*-benzyl-2,3-di-[(*R*)-3-decanoyloxytetradecanoylamino]-2,3-dideoxy-β-D-allopyranoside as a colorless oil. (10) A solution of 2-[(*R*)-3-decanoyloxytetradecanoylamino]ethyl 6-*O*-benzyl-2,3-di-[(*R*)-3-decanoyloxytetradecanoylamino]-2,3-dideoxy-β-D-allopyranoside (105 mg, 0.072 mmol) in anhydrous dimethylformamide (5 mL) was treated with sulfur trioxide triethylamine complex (78 mg, 0.43 mmol). The reaction was heated to 50 °C for 5 h. An additional amount of sulfur trioxide triethylamine complex (100 mg, 0.55 mmol) was added and the reaction stirred at 50 °C for 18 h. The reaction mixture was concentrated in vacuo. Chromatography on C_18_ column (gradient elution, 5→ 20% methylene chloride + 1% triethylamine/methanol) afforded 90 mg (82%) of 2-[(*R*)-3-decanoyloxytetradecanoylamino]ethyl 6-*O*-benzyl-2,3-di-[(*R*)-3-decanoyloxytetradecanoylamino]-2,3-dideoxy-4-*O*-sulfoxy-β-D-allopyranoside triethylammonium salt as a white salt. (11) A solution of 2-[(*R*)-3-decanoyloxytetradecanoylamino]ethyl 6-*O*-benzyl-2,3-di-[(*R*)-3-decanoyloxytetradecanoylamino]-2,3-dideoxy-4-*O*-sulfoxy-β-D-allopyranoside triethylammonium salt (70 mg, 0.045 mmol) in a mixture of 2:1 anhydrous tetrahydrofuran: methanol (5 mL) was hydrogenated in the presence of 20% palladium hydroxide on carbon (30 mg) and triethylamine (0.034 mL, 0.00024 mmol) using a Parr hydrogenator at room temperature and 50 psig pressure for 18 hours. The reaction mixture was filtered through Celite and the filtrate was concentrated under vacuum. Chromatography on C_18_ silica column (gradient elution, 5→20% methylene chloride + 1% triethylamine/methanol), the purified material was dissolved in cold 2:1 chloroform-methanol (8 mL) and washed with cold 0.1 N aqueous hydrochloride (1.6 mL). The lower organic layer was dried over anhydrous sodium sulfate and concentrated in vacuo. The residue was salted with (1 -2 equiv.) triethylamine to give 28 mg (43%) of 2-[(*R*)-3-decanoyloxytetradecanoylamino]ethyl 2,3-di-[(*R*)-3-decanoyloxytetradecanoylamino]-2,3-dideoxy-4-*O*-sulfoxy-β-D-allopyranoside triethylammonium salt as a glassy solid: ^1^H NMR (CDCl_3_/CD_3_OD): d (ppm) 7.84 (t, *J* = 5.5 Hz, 1 H), 7.55 (d, *J* = 8.0 Hz, 1 H), 7.22 (d, *J* = 9.0 Hz, 1 H), 5.27–5.23 (m, 3 H), 4.65 (br s, 1 H), 4.59–4.55 (m, 2 H), 4.26–4.21 (m, 1 H), 4.19– 4.15 (m, 1 H), 3.85–3.79 (m, 2 H), 3.73–3.70 (m, 1 H), 3.51–3.43 (m, 2 H), 3.18 (q, *J* = 7.5 Hz, 7 H, CH_2_ of triethylamine (~1.2 equiv.)), 2.62–2.19 (m, 12), 1.64–1.52 (m, 12 H), 1.37–1.26 (m, 100 H, including 10, CH_3_ of triethylamine), 0.88 (t, *J* = 7.0 Hz, 18 H); HRMS (ESI-TOF) m/z: Calcd for C_80_H_151_N_3_O_16_S [M-H]^-^ 1441.0737, found 1441.0714.

### Drugs

Fentanyl citrate, sufentanil citrate, carfentanil HCl, methadone HCl, remifentanil HCl, and buprenorphine HCl were obtained from either Boynton Pharmacy (Minneapolis, MN), Hennepin County Medical Center pharmacy (Minneapolis, MN), or Sigma-Aldrich (St. Louis, MO). Acetylfentanyl HCl was obtained from RTI International. Drug doses are expressed as the weight of the free base.

### Ethics statement

Studies were performed according to the Guide for the Care and Use of Laboratory Animals and the National Institute of Health. Animal protocols were approved by both the University of Minnesota and the Hennepin Healthcare Research Institute Animal Care and Use Committees. Animals were euthanized by AAALAC-approved CO_2_ chambers (rats) or via IV pentobarbital sodium (pigs), and all efforts were made to minimize suffering.

### Animals

For drug-challenge studies, 8–10 weeks old male Sprague-Dawley rats were obtained from Charles River Laboratories (Wilmington, MA). Rats were grouped housed under a standard 14/10 hour light/dark cycle and were given food and water *ad libitum*. For fentanyl self-administration, male and female Sprague-Dawley rats (65-75 day old on arrival; Envigo, Madison, WI) had ad lib access to water and restricted access to food (18–20 g/day) in a temperature- and humidity-controlled colony room under a reversed 12-hr light/dark cycle (experimental sessions ran during the dark phase). For mini pig studies, two-month-old Hanford miniature pigs were obtained from Sinclair Bio Resources (Auxvasse, MO). Pigs were housed 1–3 per pen under a 14/10 hour light/dark cycle and fed 1 cup of Envigo Teklad miniswine diet # 8753 daily. All testing occurred during the light phase.

### Experimental design

#### Drug-challenge studies in rats

Anti-fentanyl vaccines were administered i.m. in the hind thigh on days 0, 21, and 42. Animals received 5 μg unconjugated carrier protein (CRM) adsorbed on alum as a control, 5 μg F_1_-CRM with no adjuvant, 5 μg F_1_-CRM adsorbed to alum or 5 μg F_1_-CRM adsorbed on alum with 1 μg INI-2002 or 10 μg INI-4001 as described in each experimental section. Blood was collected via tail vein on day 49 for antibody analysis. Some rats were euthanized at 49 days for trunk blood collection to run in vitro binding assays. Rats were challenged starting on day 56 and challenged with one drug weekly until the end of the study. Challenge drugs included fentanyl (0.05 mg/kg-0.45 mg/kg), sufentanil (0.008 mg/kg), carfentanil (0.01 mg/kg), or methadone (4.5 mg/kg), all administered subcutaneously (s.c). Rats were boosted with vaccine every three weeks to maintain antibody titer levels. Vaccine efficacy in reducing opioid-induced behavioral effects were measured via hot plate test of centrally-mediated antinociception^20^ or in reducing opioid-induced respiratory depression and bradycardia using a pulse oximeter (Starr Life Sciences, Oakmont, PA)^20^ at baseline and every 15 minutes after drug challenge for one hour. A cutoff of 30 seconds was used in hot plate studies to prevent thermal injury. In some studies, naloxone (0.1 mg/kg) was given 1 hour after drug challenge to reverse opioid-induced effects. In terminal studies, rats were euthanized after the final timepoint, and blood and brain were collected for analysis by LC-MS.

#### Fentanyl self-administration (FSA) in rats

Rats were implanted with a jugular catheter under ketamine (75–90 mg/kg) and dexmedetomidine (0.25 mg/kg) anesthesia. The opposite end of the catheter exited the body between the scapulae and attached to a vascular-access harness (VAH95AB, Instech Inc., Plymouth Meeting, PA). Rats recovered from surgery for at least three days, during which catheters were flushed daily with a heparinized (30 units/ml) saline/glycerol (25%) solution and ceftriaxone (5.25 mg), and a s.c. injection of buprenorphine (0.05 mg/kg; first two days only) was given for analgesia. Over the course of the experiment, daily infusions of heparinized saline/glycerol continued and infusions of methohexital (0.1 ml of 10 mg/ml, i.v.) were given on Fridays after the session to determine catheter patency (indicated by anesthesia within 5 s). After recovery from surgery, rats were trained to self-administer fentanyl (1 µg/kg/infusion) under a fixed ratio (FR) 1 schedule during daily 120-min sessions (five days/week) using standard two-lever operant conditioning chambers (Med Associates, St. Albans, VT). After at least 10 sessions and when robust fentanyl intake was established, the FR was gradually increased to 3 over several sessions. The unit dose was then increased to 2.5 µg/kg/infusion. Initial training at the lower unit dose was intended to minimize side effects of high fentanyl intake that we have observed in some rats (e.g., pica, paw licking/chewing). After at least 10 sessions at the 2.5 µg/kg unit dose and once FSA stabilized, rats were immunized i.m. with 5 μg CRM, 5 μg F_1_-CRM adsorbed on 24 μg alum, or 5 μg F_1_-CRM and 10 μg INI-4001 adsorbed on 24 μg alum. Rats were vaccinated every 2 weeks on Fridays after their FSA session to maintain high antibody titers during the remainder of the study. The first 8 weeks (4 vaccinations) was a maintenance phase, during which FSA continued at the 2.5 µg/kg training dose. A blood sample was collected from the tail vein 1–2 weeks after the 4th vaccination to measure antibody titers. The following 6 weeks (3 vaccinations) was a dose-response phase, during which the effects of vaccination on the FSA dose-response curve were determined. During this phase, the fentanyl unit dose was changed weekly on Mondays (initially increased to 5.0 µg/kg/infusion and then reduced each week to 1.0, 0.75, 0.50. 0.25, and 0 µg/kg/infusion) as rats continued to be vaccinated. Following the dose-response determination, vaccination ceased, and rats were given access to 2.5 µg/kg unit dose to allow reacquisition of FSA.

If a catheter lost patency or was otherwise compromised (e.g., the rat pulled out its catheter), data from that week were excluded from analysis and another catheter was implanted in a femoral vein. The majority of these reimplants occurred during training before vaccination began. If a rat required a reimplant during the FSA maintenance phase of vaccination, rats continued being vaccinated until FSA was reacquired before beginning FSA dose-response phase. This resulted in one additional vaccination (five total) in one CRM control rat, two additional vaccinations (six total) in two F_1_-CRM+alum rats, and one additional vaccination (five total) in three F_1_-CRM+alum+INI-4001 rats during this phase. If a rat required a reimplant during the fentanyl dose-response phase of vaccination, vaccinations continued until baseline self-administration was recovered at the 2.5 µg/kg unit dose after the reimplant and the dose-response determinations were completed, resulting in one or two additional vaccinations in three CRM control rats, two F_1_-CRM+alum rats, and three F_1_-CRM+alum+INI-4001 rats. Another F_1_-CRM+alum+INI-4001 rat received six additional vaccinations during the dose-response phase because it took several weeks to reacquire FSA before completing the dose-response assessment.

#### Mini-pig studies

Nine pigs (*n* = 3/group) were treated as follows: (1) non-vaccinated control group, (2) F_1_-CRM (125 μg) formulated in alum (562.5 μg), and (3) F_1_-CRM (125 μg) formulated in INI-4001 (250 μg), and alum (562.5 μg). Active vaccination groups received a total of three immunizations on days 0, 21, and 42, which were administered i.m. in a volume of 0.6 mL. Serum was collected on days 7, 28, and 49 ± 3 days to determine antibody titers. On day 49 ± 3 days, each mini-pig was anesthetized with isoflurane delivered in pure oxygen, then orotracheally intubated with a cuffed endotracheal tube and breathed spontaneously under a light plane of isoflurane anesthesia. Intravenous and intra-arterial catheters were placed for drug administration and blood sampling. After instrumentation and stable plane of anesthesia were achieved, mini-pigs were challenged with a continuous IV infusion of fentanyl until they reached the study endpoint which was defined as two continuous minutes of apnea. Fentanyl was infused at a rate of 30 μg/kg/hr for 1 hour (0–60 minutes), increasing to 60 μg/kg/hr for the second hour (60–120 minutes), and then increasing to 120 μg/kg/hr for the third hour (120–180 minutes) for a total possible dose of 210 μg/kg. Various respiratory endpoints (time to apnea, respiratory rate, end-tidal CO_2_, tidal volume, and minute volume) were monitored using a Datex Ohmeda Compact S5 monitor (Clearwater, FL). Blood was collected prior to fentanyl infusion (baseline) and at multiple timepoints during fentanyl administration for serum fentanyl analysis. After animals were apneic for two continuous minutes, they were administered naloxone (10 μg/kg, IV) and monitored for respiratory recovery.

### Antibody analysis

Opioid-specific IgG and IgG subclass titers were measured via titer ELISA as previously described^26^. Briefly, 96-well ELISA plates (Costar 9018 EIA/RIA, Jackson ImmunoResearch Laboratories Inc., West Grove, PA) were coated overnight with 5 ng/well of unconjugated BSA or F_1_ hapten conjugated to BSA, and blocked the next day. Rat or pig serum was serially diluted in the appropriate wells starting at either a 1:200 or 1:50 dilution. Plates were then incubated with the following secondary antibodies: goat-anti-rat IgG-HRP (1:50,000; Cat. No. 112-035-003, Jackson ImmunoResearch, West Grove, PA), mouse anti-Porcine IgG (1:1000; Cat. No. 552554, BD Biosciences, Franklin Lakes, NJ), or mouse anti-Porcine IgG_2_ (1:1000; Cat. No. MCA636GA, Bio-Rad, Hercules, CA). For pig ELISAs, plates were incubated with a goat anti-mouse IgG Total HRP tertiary antibody (1:1000, Cat. No. 1030-05, Southern Biotech, Birmingham AL).

In vitro binding of fentanyl analogs and off-target drugs was measured using competitive ELISA as previously described^17,20^. Briefly, 96-well ELISA plates (Costar 9018 EIA/RIA, Jackson ImmunoResearch Laboratories Inc., West Grove, PA) were coated with 0.5 ng/well of F_3_-BSA in carbonate buffer at pH 9.6 overnight. The following day, plates were blocked with 1% gelatin. The appropriate drugs were then serially diluted on the plate with the following ranges: Fentanyl: 1.48 μM–2.79 pM; Carfentanil: 126 μM–238 pM; Alfentanil: 600 μM–1.12 nM; Remifentanil: 664 μM–1.2 nM; Acetylfentanyl: 150 μM–0.15 pM; Sufentanil: 0.9 mM–0.9 pM; Naltrexone: 5 mM–5 pM; Buprenorphine: 50 μM–0.05 pM; Naloxone: 5 mM–5 pM. A fixed dilution of serum (determined by titer ELISA) was added to each well of the plate and incubated for 2 hours. Plates were then incubated overnight with goat-anti-rat IgG-HRP (1:50,000; Cat. No 112-035-003, Jackson ImmunoResearch, West Grove, PA). Plates were developed using SIGMAFAST OPD substrate (Sigma-Aldrich, St. Louis, MO). If antibodies did not bind to drugs within the concentration range tested, results were reported as the highest concentration tested.

### LC-MS analysis of fentanyl concentration

Rat sera, rat brains, and pig sera were processed and analyzed as previously described^20^. Briefly, brain tissue was homogenized using Agilent ceramic beads for 3 min with a Beadblaster 24 homogenizer (Benchmark Scientific, Sayreville, NJ) at 6 m/s, then centrifuged for 10 min at 8609 × *g*. The supernatant was transferred transferred to a cryogenic tube and placed at −20 °C until time of extraction. Extraction of serum, brain supernatant, and standards was performed at 4°C. For standards, 20 μL of stock calibrator solution was added to 180 μL of fetal bovine serum, 200 μL of sample was used for extraction with 20 μL of internal standard solution added to all samples. 600 μL of cold LC-MS grade Acetonitrile was added to all tubes to precipitate proteins and then centrifuged at 8609 × *g* for 10 min. Supernatant was transferred to 96-well plate, evaporated to 200 μL on a Positive Pressure Manifold, Agilent (Santa Clara, CA), and then diluted with 600 μL with 2% phosphoric acid. Extraction was performed using Bond Elut PCX 96 round, 1 mL, 30 mg plate (Agilent, Santa Clara, CA). Cartridges were first washed with 500 μL methanol, 500 μL water, and then samples were loaded onto cartridges. Cartridges were washed in series, first using 600 μL mL of 2% formic acid followed by 600 μL 50% methanol: 50% acetonitrile. Cartridges were dried on a sample concentrator (Porvair, Norfolk, UK), placed above fresh 96 well plate to elute samples using 750 μL of 5% ammonium hydroxide in 50% methanol: 50% acetonitrile, and dried on the sample concentrator. Samples were reconstituted in 200 μL LC-MS grade water, 0.1% ammonium formate, 0.01% LC-MS grade formic acid (mobile phase A).

For bound/unbound serum analysis, serum samples were centrifuged at RT for 1 hour at 10,000 × *g* in a Nanosep filter unit (Pall Life Sciences, Port Washington, NY) with a 10 kDa cutoff to separate the free drug from antibody-bound fentanyl. Filter units were previously treated with 5% Tween-20 in distilled water for 2 hours at room temperature to minimize hydrophobic interactions and then rinsed with sterile distilled water. Filtrate (free drug) and whole serum samples were then processed and analyzed on LC-MS as described above. Protein-bound fentanyl was calculated as (total fentanyl–unbound fentanyl) and expressed as a percentage of total fentanyl.

### BioLayer interferometry (BLI) antibody avidity and kinetic assays

Antibody avidity assays were performed on an Octet Red 96e instrument (Fortebio). In vivo, serum samples (analyte) and biotinylated antigen, F_1_-Biotin, (ligand) were diluted in 10× Kinetic Buffer (Fortebio). Assays were performed by loading F_1_-Biotin onto pre-hydrated streptavidin sensors at 0.1 μg/ml (loading step 120 s) followed by 180 s baseline at 30 °C with shaking at 1000 rpm. Sensors were then moved into analyte for 120 s for association, followed by a 600 s dissociation step. Serum samples were run at three different dilutions determined by anti-fentanyl total IgG titers. Dissociation rate constants (*K*_diss_) were calculated by processing raw data using ForteBio HT analysis software version 11.1.3.50. All data were inspected for quality of fit to the calculated curve (*R*^2^ > 0.95), the response between 0.25 and 3, and residual value < 10% of the maximum response fitted to the curve.

### Data analysis

Each data point represents a measurement of a distinct animal unless otherwise noted. Statistical analyses were performed using Prism version 9.1.2 (GraphPad, LaJolla, CA). Mean antibody titers, drug concentrations, MPE% on the hot plate, heart rate, oxygen saturation, and *K*_diss_ measurements were analyzed by Brown–Forsythe test to determine differences in standard deviation. If there were no significant difference, metrics were analyzed by one-way ANOVA followed by Tukey’s multiple comparisons *post hoc* test. If there were significant differences in standard deviation, metrics were analyzed by Brown-Forsythe and Welch ANOVA with Dunnett’s T3 multiple comparisons *post hoc* test. The relationship between opioid-specific antibodies, total fentanyl administration, and time to apnea was analyzed via two-tailed Pearson correlation after the determination of normality using D’Agostino-Pearson’s test. Latency to respond, heart rate, and oxygen saturation data taken over multiple time points was analyzed via two-way ANOVA with the Geisser-Greenhouse correction and Tukey’s multiple comparisons post hoc test. Survival curve pairwise comparisons were analyzed via Mantel–Cox test.

For the FSA study, the mean number of infusions during the last three sessions at baseline and two weeks after each of the first four vaccine injections was used to assess vaccine effects on the maintenance of FSA. To assess vaccine effects on the FSA dose-response curve, the mean number of infusions during the last three sessions at each unit dose was used. The data were analyzed using mixed-model or one-way ANOVA with Geisser-Greenhouse correction followed by Holm-Sidak multiple comparison tests. To specifically assess the effects of vaccination on fentanyl’s reinforcing efficacy (i.e., motivation to consume fentanyl), exponential demand curve analysis was performed on fentanyl intake in each subject during FSA unit dose assessment as previously described^59,73^ (see below). The mean of the α (elasticity of demand), *Q*_0_ (intensity of demand), *P*_max_ (unit price at maximal responding), and *O*_max_ (maximal level of responding) parameters of the exponential curve fits to individual subject data were compared between groups via Brown-Forsythe and Welch ANOVA followed by Dunnett’s T3 tests for multiple comparisons. The α, *Q*_0_, and *O*_max_ values were log-transformed prior to analysis due to the non-normality of the data. Mean antibody titers were similarly compared between groups. All statistics were performed using Prism (version 9.1; GraphPad, San Diego, CA).

### Behavioral economic demand curve analysis

To directly measure the effects of vaccination on the reinforcing efficacy of fentanyl, exponential demand curve analysis was performed on fentanyl intake during FSA unit dose-response determinations as previously described^20,59,73^. Briefly, an exponential demand equation, log *Q* = log *Q*_0_ + *k*(*e*^-α*Q0*C^–1), was used to describe the relationship between fentanyl consumption and unit price (FR/unit dose). The dependent variable, *Q*, is the quantity consumed. The independent variable, *C*, is the cost of fentanyl in terms of the unit price. The free parameters, *Q*_*0*_ and α, are estimated from the best-fit function and refer to the maximum level of consumption at zero price (i.e., level or ‘intensity’’ of demand) and the rate of change in consumption with increases in unit price, respectively. The range of the exponential function, *k*, is a constant specifying the range of consumption in log units. The *k* value is held constant across data sets being compared (set to 3.63 in the present study) because changes in *k* impact the value of *α*. The value of *α* is inversely related to reinforcing efficacy so drugs that produce rapidly declining (elastic) demand curves have higher *α* values and lower reinforcing efficacy than demand curves with slower declining (inelastic) demand curves. In the present context, vaccines that increase α values for fentanyl consumption would be those that increase the sensitivity of FSA to price (increase elasticity of demand), suggesting that vaccination reduces the reinforcing efficacy or motivational strength of fentanyl^73^. Additional indices of demand were measured, including *P*_max_, the price at which consumption changes from relatively inelastic to relatively elastic, and *O*_max_, the maximum level of response output (i.e., active lever responding). Demand curve analysis was conducted using the exponential demand function for nonlinear regression in GraphPad Prism 9 software (GraphPad Software, Inc., La Jolla, CA). Because zero is not defined on a log scale, data from the 0 µg/kg unit dose (saline) were not included in the analysis. The *P*_max_ and *O*_max_ parameters were derived from *Q*_*0*_, *α*, and *k* using the spreadsheet application provided by Kaplan and Reed^74^.

### Reporting summary

Further information on research design is available in the Nature Research Reporting Summary linked to this article.

## Supplementary information

Supplementary Materials

REPORTING SUMMARY

## Data Availability

Data will be made available upon request to the corresponding author (Marco Pravetoni, mprave@uw.edu).
