# Supplementary material for: A TLR7/8 agonist increases efficacy of anti-fentanyl vaccines in rodent and porcine models
Source: NPJ Vaccines. 2023 Jul 24;8:107. doi: 10.1038/s41541-023-00697-9 (PMC10366150; doi:10.1038/s41541-023-00697-9)
Supplement: Supplementary file 1 — Supplementary Materials [file 41541_2023_697_MOESM1_ESM.docx]

**A toll-like receptor (TLR) 7/8 agonist increases efficacy of vaccines against fentanyl use disorders and overdose in rodent and porcine models**

Bethany Crouse, Shannon M. Miller, Peter Muelken, Linda Hicks, Jennifer R. Vigliaturo, Cheryl L. Marker, Alonso G.P. Guedes, Paul R. Pentel, Jay T. Evans, Mark G. LeSage, and Marco Pravetoni

Supplemental materials (index)

1) Figure 1: TLR7/8 agonist (INI-4001) increases vaccine efficacy against fentanyl

2) Figure 2: Parameters of vaccine efficacy in porcine model of fentanyl-induced respiratory depression

3) Figure 3: Fentanyl-specific serum IgG antibody titers at early timepoints in pigs


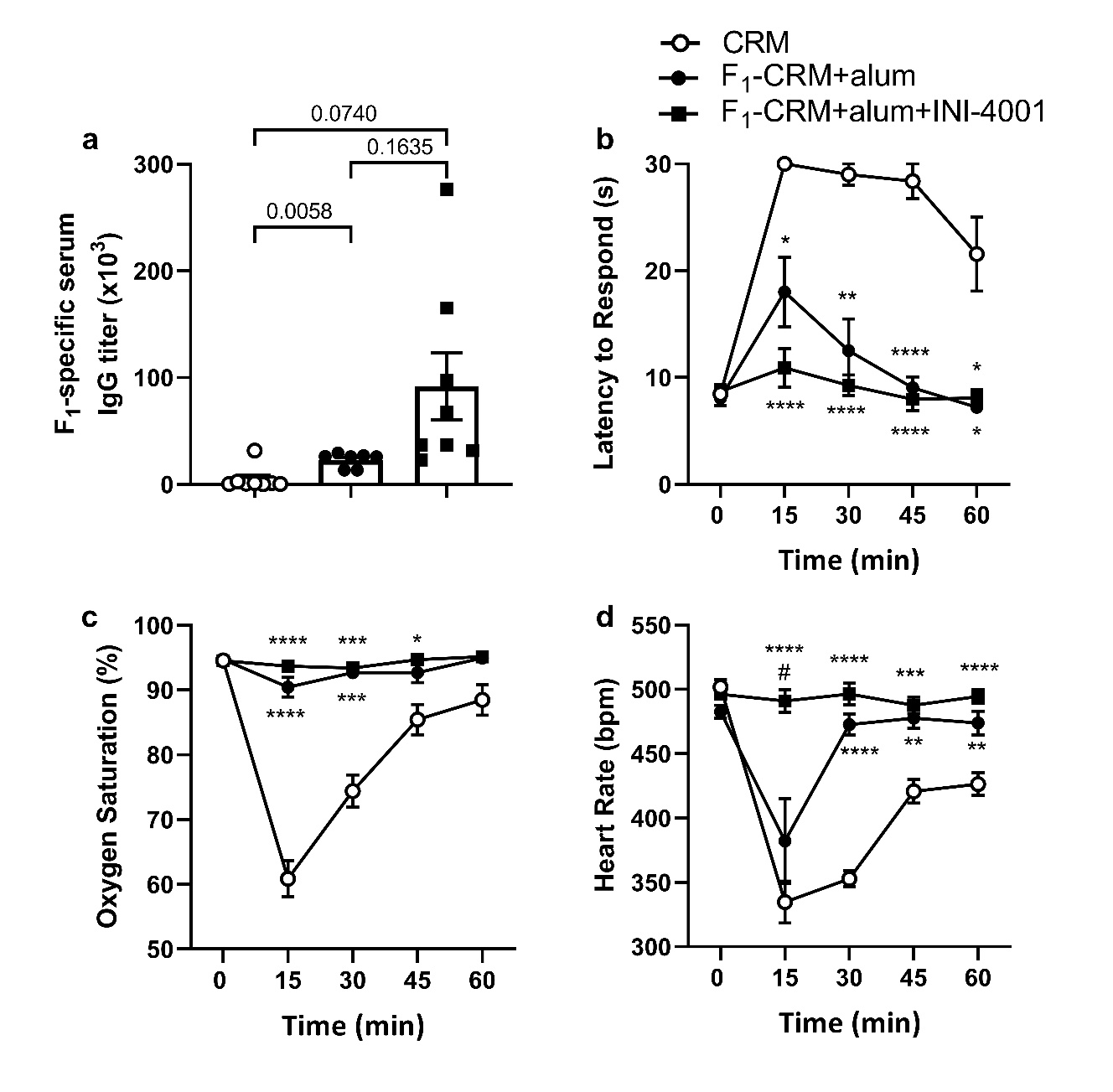


**Supplementary Figure 1: INI-4001 increases vaccine efficacy against fentanyl.** Rats were challenged s.c. with fentanyl (0.1 mg/kg) to test whether INI-4001 increased the efficacy of F_1_-CRM against fentanyl. A) Fentanyl-specific serum IgG antibody titers, measured on day 49 via ELISA. After a fentanyl challenge, B) latency to respond on a hot plate, C) oxygen saturation, and D) heart rate measured every 15 minutes over 1 hour. Data are mean±SEM. Statistical analysis via Brown-Forsythe and Welch ANOVA with Dunnett’s T3 multiple comparison’s post hoc test or two-way ANOVA paired with Tukey’s multiple comparison’s post hoc test. * directly over data points indicate significance compared to the control. # directly over data points indicate significance to F_1_-CRM+alum. Statistical symbols: * or # p<0.05, ** p<0.01, *** p<0.001, **** p<0.0001.

**Supplementary Figure 2: Parameters of vaccine efficacy in porcine model of fentanyl-induced respiratory depression**. During a fentanyl challenge, mini-pigs (n=3/group) were monitored over time for A) respiratory rate, B) tidal volume, C) end-tidal CO_2_, D) oxygen saturation, E) minute volume, and F) serum fentanyl concentrations. Data are expressed as the mean of n=1-3 animals. Error bars omitted for clarity.


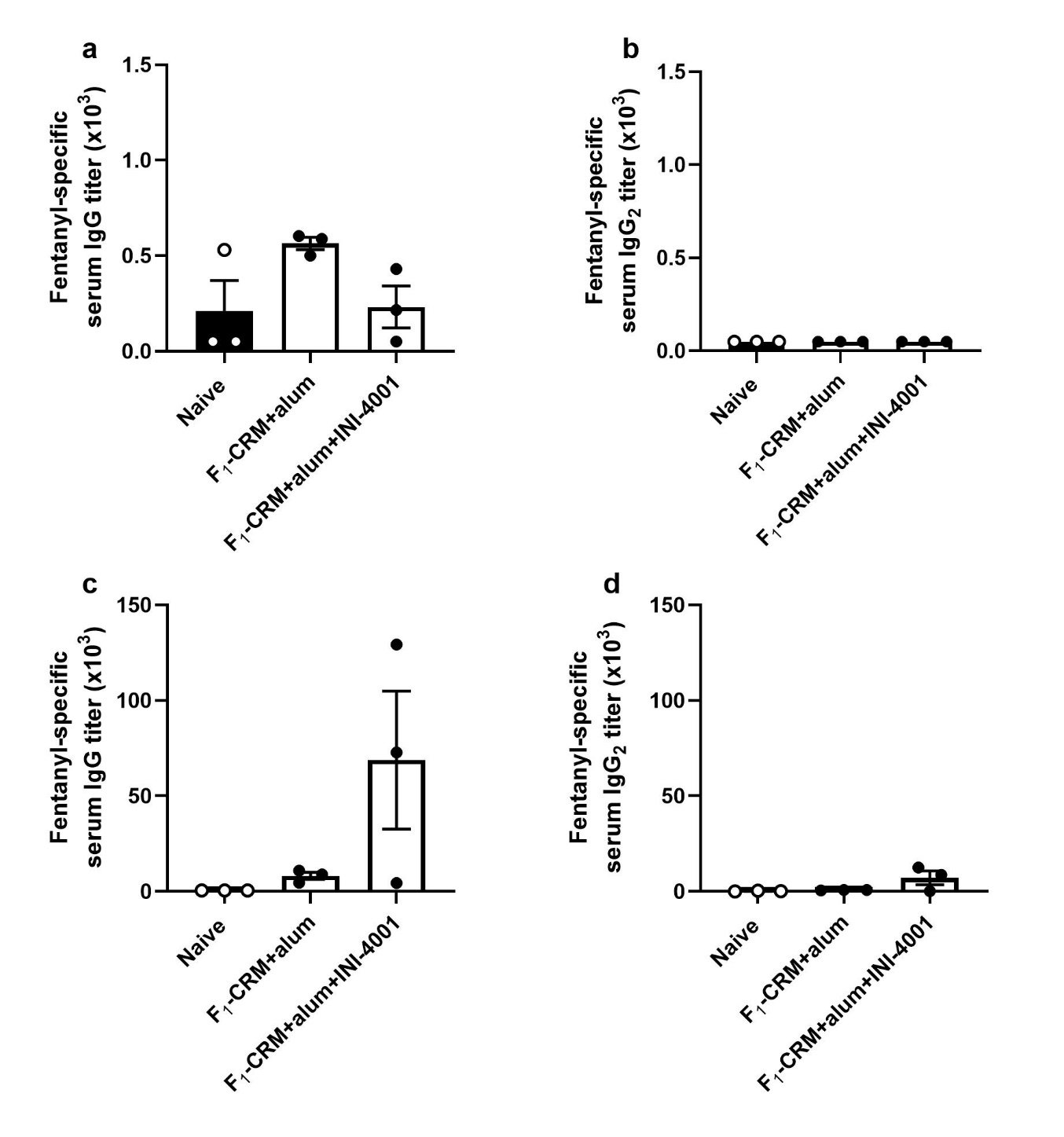


**Supplementary Figure 3: Fentanyl-specific antibody titers at early timepoints in mini-pigs.** At 7 days, fentanyl-specific serum A) total IgG or B) IgG_2_ titers measured via ELISA. At 28 days, fentanyl-specific serum C) total IgG or D) IgG_2_ titers measured via ELISA. Data are mean±SEM.
