# Supplementary material for: A TLR7/8 agonist increases efficacy of anti-fentanyl vaccines in rodent and porcine models
Source: NPJ Vaccines. 2023 Jul 24;8:107. doi: 10.1038/s41541-023-00697-9 (PMC10366150; doi:10.1038/s41541-023-00697-9)
Supplement: Supplementary file 2 — REPORTING SUMMARY [file 41541_2023_697_MOESM2_ESM.pdf]

nature portfolio

Corresponding author(s): Bethany Crouse

Last updated by author(s): Nov 17, 2022

Reporting Summary

Nature Portfolio wishes to improve the reproducibility of the work that we publish. This form provides structure for consistency and transparency in reporting. For further information on Nature Portfolio policies, see our Editorial Policies and the Editorial Policy Checklist.

Statistics

For all statistical analyses, confirm that the following items are present in the figure legend, table legend, main text, or Methods section.

n/a	Confirmed	
☐	☒	The exact sample size (*n*) for each experimental group/condition, given as a discrete number and unit of measurement
☐	☒	A statement on whether measurements were taken from distinct samples or whether the same sample was measured repeatedly
☐	☒	The statistical test(s) used AND whether they are one- or two-sided<br>*Only common tests should be described solely by name; describe more complex techniques in the Methods section.*
☒	☐	A description of all covariates tested
☐	☒	A description of any assumptions or corrections, such as tests of normality and adjustment for multiple comparisons
☐	☒	A full description of the statistical parameters including central tendency (e.g. means) or other basic estimates (e.g. regression coefficient) AND variation (e.g. standard deviation) or associated estimates of uncertainty (e.g. confidence intervals)
☐	☒	For null hypothesis testing, the test statistic (e.g. *F*, *t*, *r*) with confidence intervals, effect sizes, degrees of freedom and *P* value noted<br>*Give P values as exact values whenever suitable.*
☒	☐	For Bayesian analysis, information on the choice of priors and Markov chain Monte Carlo settings
☒	☐	For hierarchical and complex designs, identification of the appropriate level for tests and full reporting of outcomes
☒	☐	Estimates of effect sizes (e.g. Cohen's *d*, Pearson's *r*), indicating how they were calculated

Our web collection on statistics for biologists contains articles on many of the points above.

Software and code

Policy information about availability of computer code

Data collection	Mouse OxPlus software was used to collect oximetry data in rats. A Datex Ohmeda Compact S5 monitor was used to collect oximetry data in miniature pigs. MED-PC IV software was used to collect behavioral data in fentanyl self-administration studies.
Data analysis	Prism 9.1.2

For manuscripts utilizing custom algorithms or software that are central to the research but not yet described in published literature, software must be made available to editors and reviewers. We strongly encourage code deposition in a community repository (e.g. GitHub). See the Nature Portfolio guidelines for submitting code & software for further information.

Data

Policy information about availability of data

All manuscripts must include a data availability statement. This statement should provide the following information, where applicable:

- Accession codes, unique identifiers, or web links for publicly available datasets
- A description of any restrictions on data availability
- For clinical datasets or third party data, please ensure that the statement adheres to our policy

Data will be made available upon reasonable request to the corresponding author.

Human research participants

Policy information about studies involving human research participants and Sex and Gender in Research.

Reporting on sex and gender	N/A
Population characteristics	N/A
Recruitment	N/A
Ethics oversight	N/A

Note that full information on the approval of the study protocol must also be provided in the manuscript.

Field-specific reporting

Please select the one below that is the best fit for your research. If you are not sure, read the appropriate sections before making your selection.

☒ Life sciences ☐ Behavioural & social sciences ☐ Ecological, evolutionary & environmental sciences

For a reference copy of the document with all sections, see nature.com/documents/nr-reporting-summary-flat.pdf

Life sciences study design

All studies must disclose on these points even when the disclosure is negative.

Sample size	In most studies, sample size was powered from our existing data to provide an ≥80% chance of detecting differences in group means with a p value of <0.05. Studies involving pigs were pilot studies therefore they were not powered for statistical analysis.
Data exclusions	1 data point each was excluded from Figure 2g and h as outliers after running the LC/MS analysis. Data was not excluded from other figures.
Replication	Results from figure 1 were replicated in a previous mouse study (see Miller et. al dual submission), and the findings related to TLR7/8 agonist increasing efficacy were additionally replicated in Figure 2. Studies showing lack of cross reactivity (figure 3) have been extensively tested with the F1-CRM+alum formulation, and with TLR7/8 agonists in with F1-CRM and other vaccine formulations. Studies in Figures 4-6 have been replicated with F1-CRM,+alum, although some data was not consistent which is described within the text. The studies reported in this manuscript were performed with separate cohorts to increase the validity of the findings. Miniature pig studies were not replicated, as they were a pilot study.
Randomization	All animals were randomly allocated to each group.
Blinding	In rat efficacy studies, experimenters were blinded to treatment condition. In rat FSA studies, experimenters were not blinded to treatment conditions. Experimenters were not blinded during pig experiment because it was a pilot study.

Reporting for specific materials, systems and methods

We require information from authors about some types of materials, experimental systems and methods used in many studies. Here, indicate whether each material, system or method listed is relevant to your study. If you are not sure if a list item applies to your research, read the appropriate section before selecting a response.

Materials & experimental systems

n/a	Involved in the study	
☐	☒	Antibodies
☒	☐	Eukaryotic cell lines
☒	☐	Palaeontology and archaeology
☐	☒	Animals and other organisms
☒	☐	Clinical data
☒	☐	Dual use research of concern

Methods

n/a	Involved in the study	
☒	☐	ChIP-seq
☒	☐	Flow cytometry
☒	☐	MRI-based neuroimaging

Antibodies

Antibodies used	goat-anti-rat IgG-HRP, Jackson ImmunoResearch, Polyclonal, Code: 112-035-003; mouse anti-Porcine IgG, BD Biosciences, Clone

Antibodies used	F007-1241, Catalog number 552554; mouse anti-Porcine IgG2, Bio-Rad, clone K68 IgG2, Catalog number MCA636GA; goat anti-mouse IgG Total HRP Southern Biotech, polyclonal, Catalog number 1030-05.
Validation	All antibodies are commercially available and validated by the manufacturer.

Animals and other research organisms

Policy information about studies involving animals; ARRIVE guidelines recommended for reporting animal research, and Sex and Gender in Research

Laboratory animals	For drug challenge studies, 8-10 week old male Sprague-Dawley rats were obtained from Charles River Laboratories (Wilmington, MA). For fentanyl self-administration, male and female Sprague-Dawley rats were obtained from Envigo and were 65-75 day old on arrival For mini pig studies, two-month-old Hanford miniature pigs were obtained from Sinclair Bio Resources (Auxvasse, MO).
Wild animals	This study did not involve wild animals.
Reporting on sex	Male and female mice were used in this manuscripts partner manuscript (Miller et al), and we have previously not found significant differences between males and females in drug challenge studies. Therefore, we only used male mice were drug challenge studies in this manuscript. Male and female rats were used for fentanyl self-administration studies. As it was a pilot study, only male pigs were used in the pig studies.
Field-collected samples	No field samples were collected for this study.
Ethics oversight	Studies were performed according to the Guide for the Care and Use of Laboratory Animals and the National Institute of Health. Animal protocols were approved by both the University of Minnesota and the Hennepin Healthcare Research Institute Animal Care and Use Committees.

Note that full information on the approval of the study protocol must also be provided in the manuscript.
